# Crystal Structures of the **C**atalytic **D**omain of *Arabidopsis thaliana* Starch Synthase IV, of Granule Bound Starch Synthase From CLg1 and of Granule Bound Starch Synthase I of *Cyanophora paradoxa* Illustrate Substrate Recognition in Starch Synthases

**DOI:** 10.3389/fpls.2018.01138

**Published:** 2018-08-03

**Authors:** Morten M. Nielsen, Christian Ruzanski, Katarzyna Krucewicz, Alexander Striebeck, Ugo Cenci, Steven G. Ball, Monica M. Palcic, Jose A. Cuesta-Seijo

**Affiliations:** ^1^Carlsberg Research Laboratory, Copenhagen, Denmark; ^2^UMR8576 CNRS-USTL, Unité de Glycobiologie Structurale et Fonctionnelle, Université des Sciences et Technologies de Lille, Villeneuve-d’Ascq, France

**Keywords:** starch synthase, crystal structure, SSIV, GBSS, ADP, acarbose, ternary complex, phylogenetic tree

## Abstract

Starch synthases (SSs) are responsible for depositing the majority of glucoses in starch. Structural knowledge on these enzymes that is available from the crystal structures of rice granule bound starch synthase (GBSS) and barley SSI provides incomplete information on substrate binding and active site architecture. Here we report the crystal structures of the catalytic domains of SSIV from *Arabidopsis thaliana*, of GBSS from the cyanobacterium CLg1 and GBSSI from the glaucophyte *Cyanophora paradoxa*, with all three bound to ADP and the inhibitor acarbose. The SSIV structure illustrates in detail the modes of binding for both donor and acceptor in a plant SS. CLg1GBSS contains, in the same crystal structure, examples of molecules with and without bound acceptor, which illustrates the conformational changes induced upon acceptor binding that presumably precede catalytic activity. With structures available from several isoforms of plant and non-plant SSs, as well as the closely related bacterial glycogen synthases, we analyze, at the structural level, the common elements that define a SS, the elements that are necessary for substrate binding and singularities of the GBSS family that could underlie its processivity. While the phylogeny of the SSIII/IV/V has been recently discussed, we now further report the detailed evolutionary history of the GBSS/SSI/SSII type of SSs enlightening the origin of the GBSS enzymes used in our structural analysis.

## Introduction

Starch, a macropolymer composed of glucose monomers, is used for storage of both carbon and energy in photosynthesizing organisms. It can be used when needed, for example to support plant growth during the night, or as a nutrient source for seedlings (Zeeman et al., 2010; Sonnewald and Kossmann, 2013). Starch is the main source of calories in human nutrition, both directly and as animal fodder, while it also finds many industrial uses such as papermaking and biodegradable plastics (Sonnewald and Kossmann, 2013).

Starch biosynthesis is carried out by a cohort of enzymes working in concert. In cyanobacteria, green algae and land plants, ADP-glucose pyrophosphorylase synthesizes ADP-Glc which acts as the glucose donor. Starch Synthases (SSs) transfer glucose from ADP-Glc donor to elongate pre-existing glucose chains via α-1,4-linkages. Branching enzymes transfer fragments of the chains to create α-1,6-linked branches. Other enzymes are also required for normal starch synthesis, including debranching enzymes, phosphatases, dikinases, amylases, disproportionating enzymes and starch phosphorylases (Fujita, 2014; Tetlow and Emes, 2017). Interestingly unlike the SSs, the other enzymes have a common origin in Archaeplastida independently of the compartment where starch is found (for reviews see Ball et al., 2011; Ball et al., 2015).

Starch is composed of two distinct molecules: amylose, an α-1,4-linked linear polymer with few α-1,6 branches, and amylopectin, which contains numerous branching points. Starch can be defined as an “abnormal” solid and semi-crystalline glycogen-like polymer that accumulates in the form of granule aggregates of unlimited size. Amylose synthesis is carried out by granule-bound starch synthase (GBSS) while amylopectin is synthesized by soluble SSs I-V.

Glycogen, another major glucose storage polymer, is also comprised of α-1,4-linked glucose with frequent α-1,6 branches. Glycogen is widely distributed in the three domains of life, Archaea, Bacteria and Eukaryotes. The distribution of starch is more restricted; it is found in a few cyanobacterial species, in all Archaeplastida (defined here as the ancestral photosynthesizing eukaryotes red and green algae, land plants and glaucophytes) and a few eukaryotic algae and protists. These consist of the unicellular alveolates and cryptophyte algae, which are both derived from Archaeplastida through “secondary” plastid acquisition (Deschamps et al., 2006, 2008a; Curtis et al., 2012). This occurred through endosymbiosis of a red alga by another eukaryote. Despite the fact that Archaeplastida are related to cyanobacteria through the unique primary plastid endosymbiosis that introduced this organelle in eukaryotes, it appears that the ability to accumulate starch was not transmitted through cyanobacteria to the Archaeplastida ancestor. It is suggested that the transition from glycogen to starch synthesis occurred several times independently in both cyanobacteria and Archaeplastida (Ball et al., 2015).

Bacteria synthesize glycogen through the bacterial-specific glucosyl nucleotide ADP-Glc while eukaryotes exclusively use UDP-Glc as the donor for glycogen synthesis (Ball et al., 2011, 2015). At first glance starch synthesis uses the glucosyl nucleotide donor corresponding to the ancestry of the compartment where the transition of glycogen to starch metabolism occurred. This corresponds to UDP-glucose for the cytosolic starches of red algae, glaucophytes, and alveolates and also for the periplastidial starches of cryptophytes. For the green alga and plant plastidial starches, as well as for cyanobacteria, it corresponds to ADP-Glc. Hence starch is solely made in plastids in green algae and land plants while it is found exclusively in the cytosol of red algae, glaucophytes and alveolates. Glaucophytes are fresh water unicellular algae that harbor a peptidoglycan-containing plastid (the muroplast). Only a dozen species are known and *Cyanophora paradoxa* has been the most intensively studied. Among Archaeplastida, glaucophytes are believed to retain the largest set of ancestral features. We have previously reported a detailed biochemical analysis of starch structure and synthesis in this alga (Plancke et al., 2008). In cryptophytes, starch is found in the periplast (Deschamps et al., 2006), a compartment surrounding the plastid that corresponds to the cytosol of the ancient red alga that was engulfed through secondary endosymbiosis by a eukaryotic phagotroph.

Starch synthases have a central role in starch synthesis since almost every glucose found in starch was initially deposited by an SS enzyme. SSs are retaining glycosyltransferases classified within the GT5 family in the Carbohydrate Active Enzyme (CAZy) database (Lombard et al., 2013). GT5 also includes the ADP-Glc utilizing bacterial glycogen synthases (GSs). Two very different types of GT5 SS are used for starch synthesis. Red algae and glaucophytes share a GT5 UDP-Glc utilizing glycogen/SS with many heterotrophic glycogen accumulating eukaryotic lineages. Other glycogen synthesizing eukaryotes such as animals or fungi use a distinct GT3 enzyme for the same purpose and some amoebas use both GT3 and GT5 types of glycogen synthases (Ball et al., 2015). GT3 and GT5 families share fold, products and catalytic mechanism, but they are distinguishable at the sequence level. Furthermore, only the GT3 family, which uses UDP-Glc as donor, is known to be allosterically regulated (Roach et al., 2012). Green alga and plant SSs belong to a very widely distributed family of distantly related bacterial GT5 ADP-Glc specific enzymes, otherwise found exclusively in bacteria and some archaea. These green alga and plant SSs are divided into six different classes, of which granule bound starch synthases (GBSSs) are responsible for the synthesis of amylose. They have a processive mechanism in which the growing linear glucose chain (the acceptor in the reaction) is not released between consecutive reaction cycles and are physically localized inside the starch granule (Denyer et al., 1999). GBSS is also found in some unicellular red algae and alveolates, and in all glaucophytes and cryptophytes. GBSSI from the glaucophyte *Cyanophora paradoxa* has been shown to prefer UDP-Glc as substrate but to be also capable of using ADP-Glc as the sugar donor (Plancke et al., 2008).

The other five classes are soluble SSs: SSI, SSII, SSIII, and SSIV, which participate in the elongation of chains in amylopectin with different substrate preferences (Cuesta-Seijo et al., 2016); and SSV which has been recently identified and for which a function has not yet been determined (Liu et al., 2015). SSIII and SSIV appear to be specifically involved in the process of starch granule initiation or at least to control of the number of starch granules in chloroplasts (Roldán et al., 2007; Crumpton-Taylor et al., 2013; Guo et al., 2017; Malinova et al., 2017). However, the precise role of SSIV has not yet been clarified (Seung et al., 2016).

Current structural information on SSs is limited to GBSSI from rice (OsGBSSI) (Momma and Fujimoto, 2012) and SSI from barley (HvSSI) (Cuesta-Seijo et al., 2013). Both exhibit a characteristic GT-B fold with distinct N- and C-terminal Rossmann-like domains connected by a linker region. Two structures of the catalytic domain of OsGBSSI are available: an apo-structure to 2.7 Å resolution and a complex with ADP to 3.0 Å. Both rice structures feature a disulfide bridge suspected to have contributed to keeping them in the closed conformation, with both Rossmann fold subdomains close together and capable of forming a functional active site. The structure of the catalytic domain of HvSSI, also solved to 2.7 Å resolution, captured an open form of the enzyme, where the two subdomains are further apart than in the closed conformation. The barley structure has a regulatory disulfide bridge resulting in disordered active site loops. None of the SS structures provide information on the binding of acceptor α-glucan chains in the active site.

There is structural information on three other enzymes of the GT5 family. Several structures have been determined for *Escherichia coli* glycogen synthase (EcoGS), including the apo-enzyme (Sheng et al., 2009a), complexes with maltooligosaccharides including in the active site cleft (Sheng et al., 2009b), and complexes with ADP, glucose and buffer molecules acting as acceptor mimics (Sheng et al., 2009a). These include structures in both open and closed conformations. There are structures for GS of *Agrobacterium tumefaciens* in its apo form and bound to ADP in an open state (Buschiazzo et al., 2004). There are several structures of GS from the archaea *Pyrococcus abyssi* (Horcajada et al., 2006; Díaz et al., 2011, 2012). While the enzyme is capable of using both ADP-Glc or UDP-Glc, it is more distantly related to SSs. There are no structures of a GT5 enzyme with intact ADP-Glc. Attempts to do so have resulted in structures with ADP and either glucose or glucose derivatives in the active site (Sheng et al., 2009a; Díaz et al., 2012). The only structure with an acceptor bound to a GS lacks intact donor (Sheng et al., 2009b).

Here we present three new crystal structures of SSs of different families and origins: the crystal structure of the catalytic domain of SSIV from *Arabidopsis thaliana* (AtSSIV), of GBSS from *Cyanobacterium* sp. CLg1 (CLg1GBSS) and of GBSSI from the glaucophyte *Cyanophora paradoxa* (CpGBSSI). In all cases, they are in the closed conformation forming ternary complexes with ADP in the donor site and the inhibitor acarbose occupying the donor glucose and glucose acceptor sites that approximates the active site at the time of the reaction. The structure of CLg1GBSS also includes an example of the enzyme bound to ADP and glucose, with the acceptor site vacant, which illuminates the effects and consequences of the presence of bound acceptor both on the enzyme and the glucose donor.

The structures provide the conformation of the entire catalytic domain of all three enzymes, which was not available before for SSs. Furthermore they expand structural information on plant SSs to the SSIII – SSIV group, which is evolutionarily distinct from the GBSS – SSI – SSII group (Leterrier et al., 2008).

With the new structures and those previously determined, several families of isozymes with origins inside, at the edge, and outside of the plant kingdom, it is possible to ascertain the inherent properties of a SS. We analyze which structural features are maintained and which are variable for SSs and within the GT5 family of enzymes. We look in detail at interactions at the donor and acceptor sites. Furthermore, we analyze conserved and non-conserved secondary structural elements, which sheds light and creates new questions on the origin of the processivity of granule bound SSs.

## Materials and Methods

### Protein Expression and Purification

For expression of CLg1GBSS, a synthetic gene, codon optimized for *E. coli* expression, was purchased from DNA2.0. Tuner (DE3) cells were transformed with a pJexpress414 plasmid encoding CLg1GBSS with an N-terminal His_6_ affinity tag and linker (the sequence in the plasmid is given in Supplementary Information). A single colony was used to inoculate cultures of LB media containing 30 μg/mL of kanamycin. Two 1 L cultures were grown at 37°C until the optical density at 600 nm reached 0.6, at which point protein expression was induced by addition of 100 μM IPTG. The cultures were incubated overnight with shaking at 16°C. The cell pellets were collected by centrifugation and were re-suspended in 10 ml buffer A (20 mM Tris/HCl, pH 8.0, 500 mM NaCl, 40 mM imidazole, 10% (v/v) glycerol) per gram of cell pellet together with one protease inhibitor tablet (Roche). The cell suspension was lysed using a continuous cell disruptor (1.35 kBar, Constant Systems Ltd.). DNAseI and MgSO_4_ (final conc. 10 mM) were added to the cell lysate, which was incubated on ice for 15 min. After centrifugation, the filtered supernatant was loaded on a 5 ml HisTrap Crude column (GE Healthcare). The HisTrap column was washed with 10 column volumes (CV) of buffer A on an ÄKTA FPLC (flow rate, 1 ml/min). Protein was eluted with a gradient from 0 to 40% B over 10 CV (buffer B was as buffer A with 500 mM imidazole instead). The eluted fractions were mixed with DTT and EDTA, pH 8.0 (10 mM of each as final concentrations). Selected fractions were pooled and concentrated to ∼0.5 ml using a Vivaspin centrifugal filter (cutoff 30 kDa). This was diluted with buffer A (as above) to a final volume of 1.2 ml. 1 ml was injected on a HiLoad Superdex75 16/60 GL column equilibrated with 20 mM Tris/HCl, pH 8.0, 150 mM NaCl, 10% (v/v) glycerol, 1 mM DTT and 1 mM EDTA and eluted with the same buffer. Selected fractions from this column were pooled and concentrated in a Vivaspin concentrator (10 kDa cutoff). The concentration was determined to be 10.06 mg/ml using an extinction coefficient of 1,044 ml mg^-1^ cm^-1^.

For AtSSIV, a synthetic gene expressing the full-length sequence (without the transit peptide) was purchased from DNA2.0 and this, as well as a truncated sequence, were re-cloned into pET151 plasmids (exact sequences in the plasmids for the full-length protein and the crystallized fragment are given as Supplementary Information). Regions other than the catalytic domain were excluded for crystallization, as extended and flexible regions can hinder crystallization. Expression in Tuner cells proceeded as described above but inducing with 1 mM IPTG. Nickel column purification was performed with binding in 20 mM Tris-HCl pH 8, 250 mM NaCl, 10% glycerol and 40 mM imidazole followed by elution in the same buffer with 500 mM imidazole. The eluate was treated overnight at room temperature with 0.5 mL of TEV protease at 10 mg/mL, 0.5 mM EDTA and 1 mM DTT to cleave the His-tag and linker. After dialysis in 20 mM Tris-HCl pH 8, 250 mM NaCl, 10% glycerol and 10 mM imidazole, the protein was loaded again onto a Histrap column and collected as the eluate. The protein was concentrated and further purified in a Superdex200 26/60 size exclusion column and concentrated to 10 mg/mL based on the extinction coefficient essentially as described above.

For CpGBSSI, synthetic gene production, expression and purification proceeded as described for Clg1GBSS up to the point of the histidine gradient, which was to 45% buffer B. The protein was further purified by cation exchange chromatography using a ResourceS column (GE Healthcare) 20 mM MES, pH 6.0, 1 mM DTT, 10% glycerol as buffer A for equilibration and the same buffer with 1 M NaCl as buffer B for elution, with the actual gradient running from 10% to 40% buffer D. Selected fractions were pooled, concentrated and buffer exchanged into buffer A using a Centriprep spin filter with a cutoff of 30 kDa.

### Crystallization

For CLg1GBSS, 3 μL of protein at 10 mg/mL with 10 mM acarbose and 10 mM ADP was mixed with 2 μL of reservoir consisting of 2M (NH_4_)_2_SO_4_, 2% PEG400 and 150 mM HEPES buffer, pH 7.5, and 5 μL of water. This drop was incubated as a sitting drop over the reservoir at 15°C. The best crystal grew to an approximate size of 200 μm by 200 μm by 100 μm and was cryoprotected by addition of and mixing with 3 μL of 1.5 M (NH_4_)_2_SO_4_ and 2 M glucose to the hanging drop resulting in approximately 1 M glucose prior to freezing in liquid nitrogen.

For AtSSIV, a hanging drop was prepared by mixing 1 μL of 100 mM acarbose with 20 mM ADP (disodium salt adjusted to pH 7 with 1 M HCl), 2 μL of 0.5 mM ZnCl_2_, 0.5 μL of reservoir solution and 0.5 μL of protein stock at 10 mg/mL. This drop was incubated at 15°C over 0.5 mL of the same reservoir, which was 0.2 M Li_2_SO_4_, 0.1 M Bis-Tris pH 5.5, 25% PEG3350 (directly from condition #74 in Index Screen HT from Hampton Research). For cryoprotection, a crystal was briefly transferred to a 2 μL drop of a 1:1 mixture of precipitant and 200 mM acarbose and 25 mM ADP at pH 7; the crystal was then frozen in liquid nitrogen.

For CpGBSSI, 2.5 μL of protein stock at 9.97 mg/mL containing 20 mM DTT, 10 mM acarbose, 10 mM ADP, 10 mM MES pH 6.0 and 3% glycerol were mixed with 2 μL of reservoir solution consisting of 40 mM citric acid, 60 mM Bis-Tris propane, pH 6.4 and 20% PEG 3350 as well as 0.5 μL of 0.1 M chromium chloride, and incubated over the reservoir at 15°C. The best crystal was cryoprotected by dipping it into a solution containing 27% PEG 400, 18 % PEG 3350, 36 mM citric acid, 54 mM Bis-Tris propane buffer, pH 6.4, 9 mM acarbose, 9 mM ADP and 9 mM DDT, followed by plunging the crystal in liquid nitrogen.

### Data Collection, Structure Solution and Refinement

Diffraction data for CLg1GBSS was collected at ESRF, beamline ID29 with a wavelength of 0.953 Å at 100 K with a detector distance of 514.72 mm. 1200 images were collected with an oscillation angle of 0.3° per image and an exposure time of 50 ms per frame. Diffraction data was reduced with XDS (Kabsch, 2010) to a resolution limit of 2.2 Å. A test set was created with 3% of the data. Data quality as well as final refinement statistics are reported in **Table 1**.

**Table 1:** Data collection and refinement statistics for the crystals.

Protein	CLg1GBSS	AtSSIV	CpGBSSI
Space group	C_2_	P2_1_2_1_2_1_	P2_1_2_1_2_1_
Unit cell	a = 194.3 Å, b = 132.7 Å, c = 127.7 Å, β = 126.3°	a = 129.76 Å, b = 166.92 Å, c = 47.94 Å	A = 69.300 Å, b = 106.100 Å, c = 175.500 Å
Resolution range	2.20 Å (2.40 Å)	2.55 Å (2.70 Å)	2.90 Å (3.00 Å)
Completeness	94.7% (78.8%)	93.6% (84.5%)	90.8% (92.6%)
Redundancy	6.2 (4.1)	6.1 (2.8)	3.5 (3.4)
R_merge_	7.6% (131.5%)	12.4% (73.8%)	32.3 (216.8%)
I/σ(I)	14.03 (1.12)	11.93 (1.58)	4.59 (0.72)
CC_1/2_ (XDS)	99.9% (48.0%)	99.6% (60.8%)	96.8% (42.9)
R_work_	20.5%	18.3%	27.3%
R_free_	22.4%	22.6%	30.3%
Av. B factor	75.3 Å^2^	49.2 Å^2^	63.5 Å^2^
Av. B factor (ligands)	72.7 Å^2^	36.7 Å^2^	51.4 Å^2^
R.M.S.D. bonds	0.006 Å	0.012 Å	0.009 Å
R.M.S.D. angles	1.2°	1.6°	1.5°
Ramachandran outliers (%)	0%	0.4%	2.2%
PDB code	6GNF	6GNE	6GNG

*Numbers in brackets are for the high resolution shell. #Rmerge = ΣhklΣi|Ii(hkl)- < I(hkl) > |/ΣhklΣi Ii(hkl). +Rwork = Σhkl|| Fobs|-| Fcalc||/Σhkl|Fobs|. §Rfree = Σhkl|| Fobs|-| Fcalc||/Σhkl|Fobs| calculated using a random set containing approximately between 1000 and 1500 (3% or 3647 for CLg1GBSS) of the reflections that were not included throughout structure refinement.*

For AtSSIV, data was collected at MAXlab beamline 911-3 with a wavelength of 1.000 Å at 100 K with a detector distance of 291.91 mm. 180 images were collected with an oscillation angle of 1.0° per image and an exposure time of 15 s per frame. Diffraction data was reduced with XDS to a resolution limit of 2.55 Å excluding rings from 3.795 to 3.694 Å and from 3.62 to 3.55 Å due to the presence of mild ice rings in the diffraction images. A test set was created with 3% of the data. Data quality as well as final refinement statistics are reported in **Table 1**.

Data for CpGBSSI was collected at ESRF beamline ID23-2 with a wavelength of 0.873 Å at 100 K with a detector distance of 306.5 mm. 230 images were collected with an oscillation angle of 1.0° per image and an exposure time of 2 s per frame using the helical crystal positioning mode. Selected images were combined for processing with XDS with a resolution limit of 2.9 Å. A test set was created with 3% of the data. Data quality as well as final refinement statistics are reported in **Table 1**.

The structure of CLg1GBSS was solved by molecular replacement with MOLREP (Vagin and Teplyakov, 1997) using a truncated version of the structure of GBSS from rice (PDB_ID: 3VUE) (Momma and Fujimoto, 2012) as the search model. The structure of AtSSIV was solved similarly using a truncated version of the structure of EcoGS (PDB_ID: 2QZS) (Sheng et al., 2009a) as the search model. For CpGBSSI, the molecular replacement models were the N-terminal domain from 3VUE and the C-terminal domain from 2QZS mentioned above.

Refinement of CLg1GBSS was done with REFMAC (Murshudov et al., 2011) using three TLS groups (one per protein monomer) and isotropic refinement with implicit hydrogens and local NCS restrains relating the different monomers to each other. Manual model building was done with COOT (Emsley et al., 2010). AtSSIV was refined similarly but employing only 2 TLS groups. CpGBSSI was refined similarly but with an isotropic atom model without the use of TLS and with resolution truncated from 20 to 2.95 Å for refinement.

Figures were rendered with PYMOL.^1^ Structural superpositions were performed with the secondary structure matching algorithm in COOT (Krissinel and Henrick, 2004). Sequence comparisons were done with NCBI BLAST (Johnson et al., 2008), sequence alignments were made with MAFFT (Katoh et al., 2017) using the G-INS-I algorithm with an “unalign” level of 0.8 and interpreted with ESPript 3.0 (Robert and Gouet, 2014). Secondary structure assignments were made with Stride (Heinig and Frishman, 2004).

### Phylogenetic Techniques

Sequences were retrieved using homology searches by BLAST (Altschul et al., 1997) against sequences from *Arabidopsis thaliana* SSI, SSII, GBSS and GBSS from *Cyanobacterium* sp. Clg1 and *Cyanophora paradoxa*. All sequences inside the 2000 first blast hits with an E-value less than 1e-10 were selected. We then aligned these sequences using MAFFT with the fast alignment settings (Katoh et al., 2017). Block selection was then performed using BMGE with a block size of 4 and the BLOSUM30 similarity matrix. Preliminary trees were generated using Fasttree (Price et al., 2010) and ‘dereplication’ was applied to robustly supported monophyletic clades using TreeTrimmer (Maruyama et al., 2013) in order to reduce sequence redundancy. For each protein, the final set of sequences was selected manually. Proteins were re-aligned with MUSCLE (Edgar, 2004), block selection was carried out using BMGE (Criscuolo and Gribaldo, 2010) with a block size of four and the matrix BLOSUM30, and trees were generated using IQ-TREE (Nguyen et al., 2015) under the LG4X model (Le et al., 2012) with 100 bootstrap replicates.

## Results

The crystal structures of three SSs were determined and refined. Crystallization was carried out in the presence of ADP and acarbose; these ligands are present in the final models. Acarbose, a glucosidase inhibitor used clinically for treating diabetes, was used as an acceptor mimic to obtain ternary complexes of both donor and acceptor (O’Reilly et al., 1999; Errey et al., 2010). Crystallographic statistics and PDB accession codes on all the models are shown in **Table 1**. The model for CpGBSSI is of considerably lower crystallographic quality compared to the other two.

**FIGURE 1:**
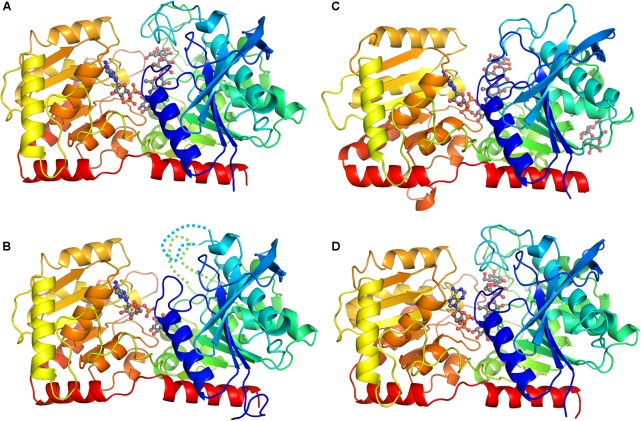
Overall structure of representative chains from each crystal. The proteins are represented as ribbons with colors from dark blue at the N-terminus through to red at the C-terminus. Ligands (except water and sulfates) are shown in ball and stick with gray carbons. **(A)** Structure of chain A in the CLg1GBSS crystal, with ADP and acarbose bound and no missing loops. **(B)** Structure of chain B in the CLg1GBSS crystal, with ADP and glucose bound; loops missing in the model are depicted as dashed lines. **(C)** Structure of chain A in the AtSSIV crystal with ADP, acarbose and a surface maltose. **(D)** Structure of chain A in the CpGBSSI crystal, with ADP and acarbose bound.

The crystal of CLg1GBSS contains three crystallographically independent protein molecules in the asymmetric unit of which one (chain A) is bound to ADP and acarbose, one (chain B) is bound to ADP and glucose from the cryoprotectant and the third chain appears to be a mixture of the two, although ADP and acarbose were modeled in the electron density. The overall structure of chains A and B is shown in **Figures 1A,B**. The overall structure reveals the characteristic GT-B fold with distinct N-terminal and C-terminal Rossmann fold domains, an interdomain linker and a crossover helix at the C-terminus linking to the N-terminal subdomain (the same arrangement is found in the other two crystals analyzed here). Two loops were disordered and not modeled in chain B, and its glucose is bound in a position equivalent to that of the last hexose, the amino-pyranose, of acarbose in chain A. The crystals of the catalytic domain of AtSSIV (**Figure 1C**) and of CpGBSSI (**Figure 1D**) both have two crystallographically independent SS molecules in their asymmetric units. In both cases both protein molecules are, as for CLg1GBSS, in the closed conformation and bound to ADP and acarbose and modeled without any missing loops.

Acarbose is bound with its non-reducing end hexose (4-amino-4,6-dideoxy-D-glucopyranose, from now on called amino-pyranose) in close contact with the phosphate moieties of ADP. Amino-pyranose mimics the glucose transferred from the donor in the reaction, while the other three acarbose hexoses occupy the binding sites of the acceptor glucan chain. All three structures are representative of the conformation of the productive ternary complex between enzyme, sugar donor and acceptor despite the fact that ADP is a reaction product.

### ADP Binding

**FIGURE 2:**
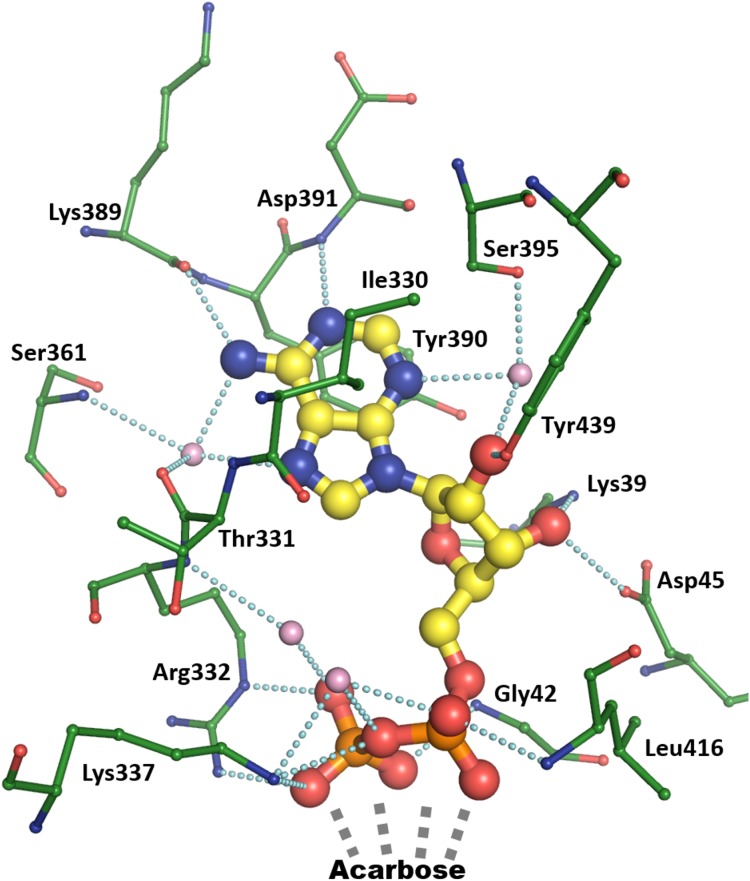
Binding of ADP by chain A of the AtSSIV structure. ADP is depicted in thick ball and stick with yellow carbons, bound amino acids are depicted in thin ball and stick with green carbons, while bound waters are pink. Dashed lines show contacts shorter than 3.2 Å.

The ADP binding mode in the AtSSIV structure will be described to serve as a reference for the other structures. AtSSIV binds ADP through an extensive network of interactions, depicted in **Figure 2**. The adenine is held in place by several short interactions, with N1 3.1 Å from the amide nitrogen of Asp391, N3 binding the side chain of Ser395 via an intermediate water, N6 binding the carbonyl of Lys389 and N7, as well as N6, having water mediated interactions with the main chain amide of Ser361 and the main chain carbonyl of Thr331. Every hydrogen bonding opportunity in the adenine is utilized for binding. It is further bound via a stacking interaction with the side chain of Tyr390 and a hydrophobic interaction with the side chain of Ile330. While the adenine is bound exclusively to the C-terminal domain of SSIV, the rest of the molecule also interacts with the N-terminal domain. The two hydroxyl groups in the ribose are involved in four short interactions: water mediated to Ser395 as for the adenine, and to the hydroxyl of Tyr439, the side chain nitrogen of Lys39 and the carboxylate of Asp45. The ether oxygen is not involved in any interactions with the protein, while O5, linking to the phosphates, is 3.1 Å away from the amide nitrogen of Gly42 but in an unfavorable orientation to form a hydrogen bond. The phosphate groups, with the distal phosphate tucked back toward the adenine, are involved in an extensive network of interactions: To the main chain nitrogens of Gly42 and Leu416 and to side chain nitrogens of Arg332 and Lys337, with two short contacts to each side chain, plus water mediated to the main chain nitrogen of Arg332 and again to the side chain nitrogen of Lys337. Further short contacts are made to the amino-pyranose of acarbose, with the distal phosphate in close proximity to the anomeric carbon around which the reaction would take place.

In comparison to AtSSIV, the number of interactions to ADP is reduced in the previously reported OsGBSSI structure and the new Clg1GBSS and CpGBSSI structures. The structure of rice GBSSI (Momma and Fujimoto, 2012), bound to ADP but not to acceptor, forms the stacking interactions, with Phe463 in the place of the tyrosine, but otherwise only the interaction between N6 and the main chain carbonyl of a lysine is present. The water bridged further interactions of N6 and N7 are missing in the structure, while N1 fails to make an interaction with a main chain amide, which is pointing away from it. The water-mediated interaction of N3 is also missing as its binding partner is now an alanine, which does not offer the possibility of a side chain hydrogen bond. Similarly, the ribose is only making one hydrogen bond to the protein via O2, with the residues which interacted with it in AtSSIV now more than 5 Å away. The phosphates are in a different conformation in the rice structure, extending away from the adenine in the absence of an acceptor. It is unclear if the reduced interactions are inherent to the rice enzyme or a result of a slightly more open conformation in the absence of bound acceptor.

CLg1GBSS, in its acceptor bound chain A, binds ADP in a way not dissimilar to that in the rice GBSS, forming only a subset of the interactions present in AtSSIV and which have already been shown in **Figure 2**: while the stacking interactions and the direct one from N6 are maintained, the water mediated ones from N6 and N7 are now reduced to the main chain nitrogen, with the carbonyl now 3.4 Å away. Similarly, N1 is now 3.4 Å away from a main chain nitrogen and N3 is missing its binding partner as the serine is again substituted by an alanine. Similarly, in the ribose only one interaction is maintained, via O2, while O3 is now 3.8 and 4.3 Å away from binding partners equivalent to those in AtSSIV. The phosphates, in contrast, are bound in a way fully equivalent to that in AtSSIV. Binding of the adenine and ribose is almost equivalent in chain B of CLg1GBSS even in the absence of acceptor, while the phosphates adopt an extended conformation, although much less extended than in the rice GBSS structure, where a glucose was not bound in the acceptor site.

**FIGURE 3:**
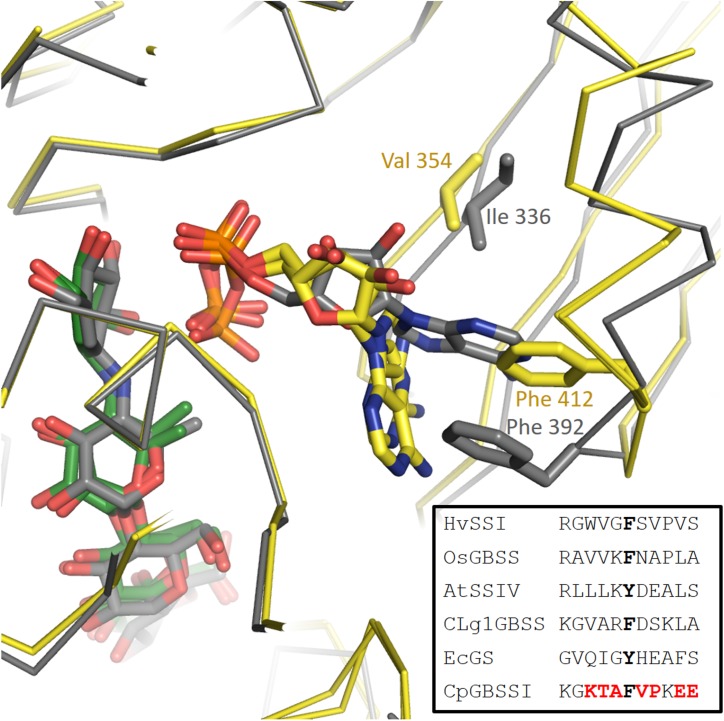
Different orientation of the adenine of ADP in the CpGBSSI structure compared to CLg1GBSS. Shown are chain A of CLg1GBSS (gray carbons and gray alpha carbon trace) and, superimposed on it, both chains of CpGBSSI (yellow carbons and yellow alpha carbon trace). The bound ADP molecules are shown with the same color scheme, while acarbose is also shown with gray carbons for CLg1GBSS and with green carbons for CpGBSSI. Residues normally involved in hydrophobic interactions with the adenine are shown including the side chain and labeled. In the inset, sequence alignment around the phenylalanines in the picture. Residues equivalent to them, which do stack with the alanine, are bolded in black. Amino acids of CpGBSSI which belong to a different functional class than their equivalents in all of the other five structures are bolded in red color.

The situation regarding ADP binding is very different for CpGBSSI, which is bound to acarbose with the adenine rotated almost 90 degrees and the ribose rotated slightly, although the phosphates make interactions fully equivalent to those in AtSSIV. The binding mode is shown in **Figure 3**. The adenines are rotated toward the acceptor molecule compared to all other structures. The adenine is neither forming any hydrogen bonds (contacts less than 3.2 Å) nor tight stacking interactions, and consequently displays some mobility and slightly different conformations in both chains in the crystal. Essentially, the adenine simply occupies an empty space in the structure. While there is a valine in the same position as that forming a hydrophobic interaction to adenine in the other structures, it is no longer capable of forming that interaction after the rotation of the adenine. The aromatic residue that makes the stacking interaction to adenine in the other structures has shifted by approximately 4 Å and it is approaching the adenine head on instead of through its pi electron cloud. It is physically occupying the position that the adenine occupies in the other structures, thus its conformation is fully incompatible with the normal adenine binding mode. The amino acids surrounding this phenylalanine are generally of different functional categories compared to those in the other five structures, as shown in the **Figure 3** inset.

### Acarbose Binding

**FIGURE 4:**
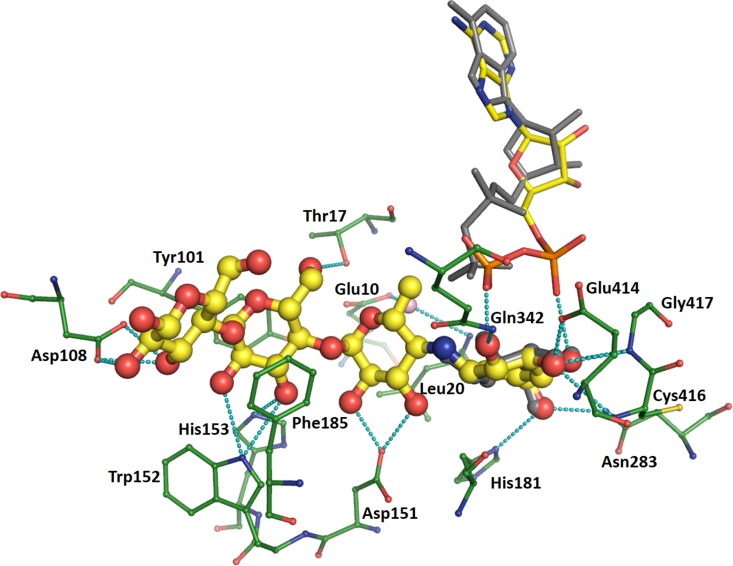
Binding of acarbose in chain A of the CLg1GBSS structure. Acarbose is shown in ball and stick with yellow carbons, its bound ADP as thinner sticks without balls and with yellow carbons, bound amino acids are shown as thin ball and sticks with green carbons and a bound water is depicted as a pink sphere. Cyan dashes depict interactions at 3.2 Å or less unless described otherwise in the main text. ADP bound to chain B (gray sticks) and the glucose bound to chain B (gray ball and sticks) are also shown after superposition of chain B onto chain A. The glucose is only partially visible as it overlaps almost perfectly with the hexose at the non-reducing end of acarbose.

Acarbose is bound similarly in all three structures, via numerous interactions which reduce in frequency from the amino-pyranose toward the reducing end of the molecule. This description will take chain A of the CLg1GBSS structure as the example (**Figure 4**) focusing on interactions at distances less than 3.2 Å. The amino-pyranose moiety at the non-reducing end is bound to the protein and the phosphates of ADP by at least two interactions from each of the hydroxyl groups. O4 is bound to the nitrogen of Gly417 and the proximal phosphate of ADP, while O3 is held in place by the carboxylate of Glu414 but also has two interactions at 3.2 Å each, just a rounding error above the cutoff chosen here, to the main chain nitrogens of Cys416 and Glu417. O2 interacts with the main chain nitrogen of Gly432 and with the distal phosphate, while O6 is bound to side chain nitrogens of Asn283 and His181. The glucose in chain B of the CLg1GBSS structure is bound in an equivalent position, overlapping with the amino-pyranose almost perfectly, and makes identical interactions with the exception of those to the phosphates, as ADP adopts a different conformation in chain B of the structure. The glucose in chain B supports this locus as that which is occupied by the glucose in ADP-Glc.

Regarding hexoses in positions corresponding to an acceptor molecule, the one carrying the methyl group has O5 bound to a water which mediates interactions to the carboxylate of Glu10 and the main chain nitrogen of Leu20, while both O2 and O3 interact with the same oxygen in the carboxylate of Asp151. While C6 is not carrying a hydroxyl group in acarbose, it would do so in a glucan acceptor molecule; in that case, that would be at a favorable distance for interactions with the distal phosphate, with the side chain nitrogen of Arg338 (not depicted in **Figure 4**) and with the carbonyl of Thr17. The third hexose is stacked on both sides of the glucosyl moiety, against the side chains of Tyr101 and Phe185. It is also oriented via interactions from O6 to the hydroxyl of Thr17 and from O3 to the side chain of His153. Its O2 and O3 also have contacts to the side chain nitrogen of Trp152, but both are at 3.3 Å. The glucose at the reducing end of acarbose has O2 and O3 interacting with both carboxylate oxygens of Asp108 while O3 is also bound to a sulfate in the crystal structure. The interaction with sulfate is likely an artifact of crystallization and is not found in any of the other structures.

Only minor differences are found in the way acarbose is bound in the AtSSIV structure and only these will be mentioned here. In the AtSSIV structure, O3 in the amino-pyranose moiety is closer than 3.2 Å to both main chain nitrogens, while O6 appears bound to the side chain oxygen rather than the nitrogen of an asparagine. The assignment of which atom is the oxygen and which is the nitrogen in an asparagine is, in this case, ambiguous from a crystallographic point of view, thus this contact should be considered as equivalent. O2 shows an extra water mediated contact linking it to the side chains of Gln336 and Arg332. Only a small displacement of the ether oxygen is worth mentioning in the second hexose: it is now 3.3 Å away from its water partner. The third hexose, here stacked between two tyrosines, has its O6 oriented differently since the threonine bound to it in CLg1GBSS is substituted in AtSSIV by a valine which cannot make side chain hydrogen bonds. Small shifts result in O3 binding to a glutamine which substitutes for the histidine in CLg1GBSS, while O2 is closer to the tryptophan and featuring new water mediated interactions to the just mentioned glutamine and Asp132. Lastly, the reducing glucose is missing the interaction with an aspartate described for CLg1GBSS, as AtSSIV is missing an extra loop present in that region in CLg1GBSS.

In the CpGBSSI structure acarbose is bound in essentially the same way. O3 in the amino-pyranose is now more than 3.5 Å away from the main chain nitrogens, while O6 also is further distant from its histidine binding partner. In the second hexose, there is no water to bind to O5, but this could be a side effect of the lower resolution of this crystallographic model, while O2 is no longer bound to an aspartate, which allows O3 to make a tighter interaction with it. The third hexose has O3 shifting away from the histidine but closer to the tryptophan. In the reducing end glucose, which is somewhat rotated compared to the other structures, O2 is no longer bound to an aspartate while O3 moves closer to it, while the anomeric oxygen has a new binding partner in the carboxylate of Asp241, which is not available in any of the other structures. In any case, it must be kept in mind that the model of CpGBSSI is of lower quality than the others described here. Also, some of the differences with CLg1GBSS are partially reversed when analyzing chain A of the CpGBSSI model, although chain A suffers from a higher degree of crystallographic disorder than chain B analyzed above.

### Conformational Changes in Response to Acceptor Binding

**FIGURE 5:**
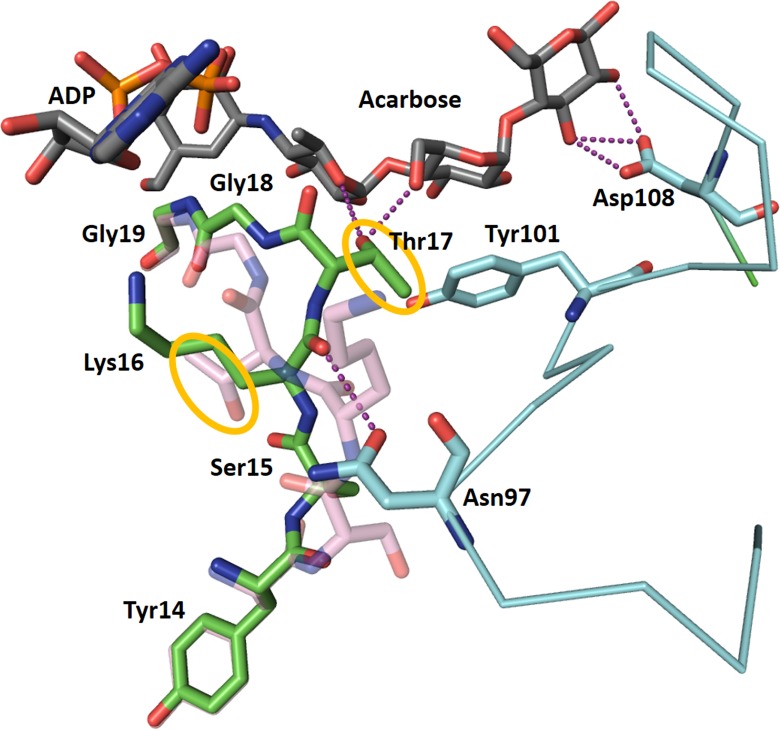
Changes in the conformation of CLg1GBSS accompanying acceptor binding. ADP and acarbose from chain A of the CLg1GBSS crystal, are shown as sticks with gray carbons. The conformation of chain A from Tyr14 to Gly19 is shown as a stick model with green carbons, while the conformation of the same segment in chain B is shown semi-transparent and with pink carbons. The loop around Tyr101 is shown as a cyan thin ribbon for the α-carbon trace with key amino acids as sticks with cyan carbons. The whole loop is disordered and absent from the model for chain B. Purple dashes represent interactions at less than 3.2 Å. Tyr101 is also participating in a stacking interaction with a glucosyl moiety in acarbose. The orange circles highlight the movement of the side chain of Thr17 upon acarbose binding.

The structure of CLg1GBSS has, in the same crystal, a protein chain (chain A) bound to ADP and acarbose and a protein chain bound to ADP and glucose (chain B). These could be described as structures in the presence and absence of acceptor. Changes in the conformation of the protein accompany acceptor binding: The loop between Ser15 and Gly18 adopts different conformations in both cases (**Figure 5**), with the largest movement corresponding to Thr17, whose hydroxyl group moves 7.1 Å to bind to the acarbose. Residues up to Tyr14 and from Gly19 in both chains adopt the same conformation. The loop between Lys92 and Asp108 is disordered and absent from the model in chain B. In chain A, in the presence of acceptor, it adopts an ordered conformation with Asn97 bound to the main chain carbonyl of Lys16, with Tyr101 forming a stacking interaction with the second glucose in acarbose and with Asp108 binding the glucose in the reducing end of acarbose. At the same time, the loop between Phe213 and Pro231 also becomes ordered, albeit with high B factors, in the presence of the acceptor, while it is disordered in chain B and absent from the final model. This loop comes in close contact, although without direct interactions, with the 92–108 loop. Chain C has properties intermediate between chain A and B. ADP in chain C was modeled in both conformations, that from chain A and that from chain B, present with partial occupancy, while acarbose has been modeled but the B factors for the amino-pyranose moiety are smaller than for the rest of the molecule, suggesting that the electron density corresponds to a composite of acarbose bound and glucose bound copies of chain C. Similarly, the loop around Tyr101 has been modeled but again with higher B factors than most of the protein chain, suggesting only partial occupancy, while the 213–231 loop could not be modeled at all. All of these features are compatible with chain C representing a mixture of the conformations and binding states present in chains A and B.

### Other Structural Features

**FIGURE 6:**
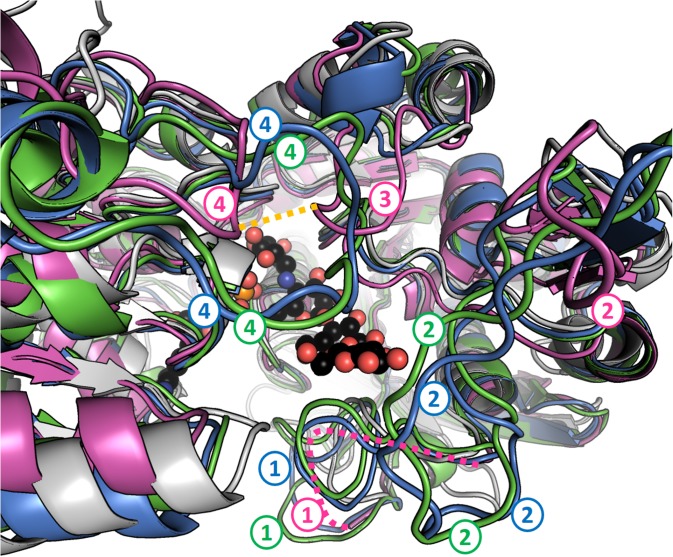
Granule bound starch synthases have extra loops in the region of the reducing end of acarbose. Perspective has been exaggerated and fog applied to better represent distance from the camera. AtSSIV is shown in white, OsGBSSI in purple, CLg1GBSS in blue and CpGBSSI in green. ADP and acarbose are shown as sphere models with black carbons from chain A of CLg1GBSS. Four regions, numbered from the N-terminus to the C-terminus, contain extra loops (compared to AtSSIV) that approach the reducing end of acarbose. Loop 1 is present at the sequence level in all three GBSSs but it is disordered in the crystal structure of OsGBSSI (depicted as a pink dashed line). Loops 2 and 4 are present but shorter in the rice protein while loop 3 is extended only in the rice structure, not as a result of an extension in the sequence, but as a result of an extended conformation where the other proteins have helices. A disulfide bridge is formed between loops labeled 3 and 4 in OsGBSSI, which is shown as a dashed orange line.

None of the three structures has disulfide bridges. Both GBSS structures described here feature a set of extra loops compared to AtSSIV. These loops, coming into proximity with each other, approach and almost surround the reducing end of the acarbose molecule. They are described and compared to their equivalents in OsGBSSI in **Figure 6**.

The prolines just before the conserved KxGGLGDV motif, which is involved in donor binding in the active site, are all *cis*-prolines. This observation is extended to the previously available structures of OsGBSSI (Momma and Fujimoto, 2012), SSI from barley (Cuesta-Seijo et al., 2013) and GS from *E. coli* (Sheng et al., 2009a,b).

The main chain carbonyl of a histidine in a strictly conserved His-Asn motif stands out in the structures. It is His181 in CLg1GBSS, His199 in CpGBSSI and His190 in AtSSIV (Supplementary Figure 1A). These carbonyl oxygens are approximately 3.5 Å above the anomeric carbon in the glucose to be transferred and directly opposite, from the perspective of that carbon, to the distal phosphate of ADP, which would be the leaving group. It has no possibility of forming any hydrogen bonds in that position. Also, the peptide bond between these histidines and the following asparagines is somewhat distorted, in the border of the allowed region in a Ramachandran plot (Chen et al., 2010) in all copies of all molecules. These observations also extend to the structures from rice, barley (even in the absence of bound ligands) and *E. coli*. All this suggests a possible involvement in the reaction, where it is placed such as to stabilize the oxocarbenium-like anomeric carbon in the transition state which has been proposed (Lairson et al., 2008; Ardévol and Rovira, 2015).

One new surface binding site for sugars is identified in the AtSSIV structure. Trp172 and Phe237 form an extended and slightly curved aliphatic patch in the surface of the protein, far away from the active site. In the case of chain A, electron density is present next to it as a flat ribbon, which is compatible with a bound acarbose molecule. Fitting of a whole acarbose was unstable and a maltose was modeled there as a generic α-glucan, but the high B factors result in a relatively feature-less density where it is impossible to ascertain the correct geometry of the bound sugars (Supplementary Figure 1B). The same area in chain B has a symmetry equivalent of chain A nearby, which blocks the possibility of glucan binding.

### A Possible Surface Binding Site Is Identified in CLg1GBSS

Tyr164 and Tyr165, on the surface of the protein, also present a similar, planar, slightly curved extended aliphatic surface which is typical of α-glucan binding sites (Cockburn et al., 2014, 2015). In the case of chain A, amino acids from the His_6_ purification tag at the N-terminus of CLg1GBSS are found bound to that surface, corresponding to a symmetry equivalent of chain C. In the case of Chain C, the His-tag of a different symmetry equivalent is bound there, in this case originating from chain B. Interestingly, only the His-tags of chains B and C are ordered in the crystal, that of chain A is disordered and hence unmodeled. In the case of chain B, this double tyrosine site has residual electron density bound to it featuring two planar blobs, each approximately 4 Å apart from each aromatic residue and parallel to them. The density’s features are too diffuse and generic to model anything there (though a maltose was compatible with it) and thus it was left empty in the final model. The strongly hydrophobic nature of this site, illustrated by the very unusual ordered binding of purification tags, and its generic geometry, typical of α-glucan binding sites, suggests that this could behave as a polysaccharide binding site *in vivo*.

### Structural Conservation Between Starch Synthases

**FIGURE 7:**
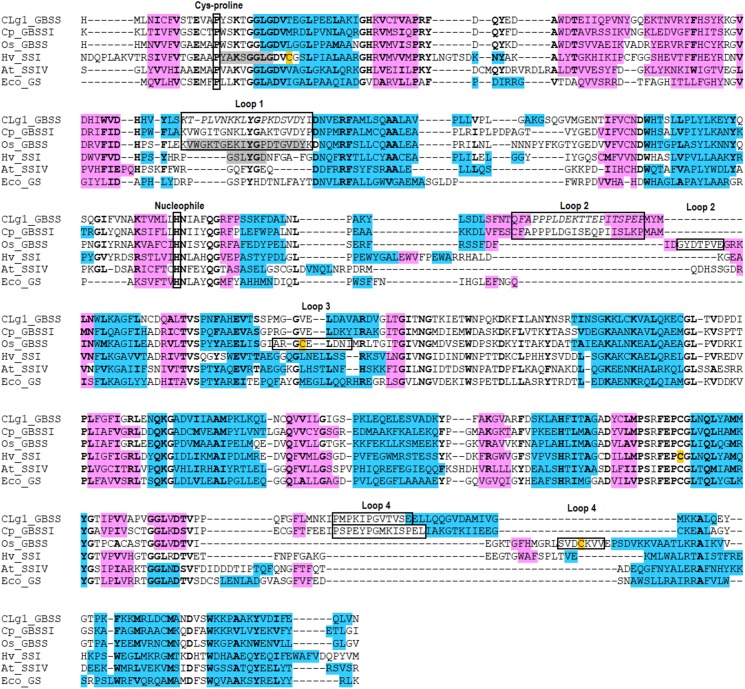
Alignment of secondary structure elements for all five SS enzymes with available structures including EcoGS. α-helices are blue, β-sheets are pink. Cysteines known to be involved in disulfide bridges are orange. Residues which are disordered in their respective structures are gray, while residues which are disordered only in some chains in the CLg1GBSS structure are in italics. Residues which show 100% conservation within one of the functional families as defined in ESPript 3.0 are bolded. Several features mentioned elsewhere in the text are shown in labeled boxes.

Overall, the structures adopt the double Rossmann fold of GT-B glycosyltransferases, with both domains coming together to form the catalytically competent conformation. Internal variation between the different chains present in each structure were small with internal R.M.S.D.s of 0.37, 0.23 and 0.32 Å for CLg1GBSS, of 0.23 Å for CpGBSSI and of 0.30 Å for AtSSIV. Conservations at the sequence level and conformationally at the R.M.S.D. level are shown in **Table 2**. The table includes EcoGS which also belongs to the GT5 family of glycosyl transferases and for which structures with donor and acceptor bound are also available. The secondary structural elements are strongly but not strictly conserved, both in terms of order and length. A sequence alignment showing secondary structure elements is shown in **Figure 7**, also depicting elements mentioned elsewhere in the text.

**Table 2:** Sequence and structural conservation between the different available structures of SSs and EcoGS.

	CLg1GBSS	CpGBSSI	OsGBSSI	HvSSI	AtSSIV	Eco GS
CLg1GBSS		1.71 Å	1.53 Å	3.36/1.43 Å	1.87 Å	1.50 Å
CpGBSSI	47%		1.90 Å	3.48/1.16 Å	1.81 Å	1.73 Å
OsGBSSI	43%	44%		3.47/1.39 Å	2.37 Å	1.74 Å
HvSSI	35%	36%	38%		3.94/1.94	3.43/1.80 Å
AtSSIV	29%	31%	30%	31%		1.75 Å
EcoGS	29%	32%	33%	33%	31%	

*For CLg1GBSS, CpGBSSI and AtSSIV, chain A has been considered from each crystal. Data from OsGBSSI is taken from PDB_ID:3VUF. Data for EcoGS is taken from PDB_ID:2QZS. Numbers in % are sequence identities after alignment with NCBI BLAST and numbers in Å are α-carbon R.M.S.D. differences calculated with Coot. In the case of HvSSI, the two different R.M.S.D. values correspond to considering the open conformation of the whole enzyme or the N-terminal domain only.*

### Kinetic Data

Kinetic data, measured chromogenically as described in (Cuesta-Seijo et al., 2016), is presented in Supplementary Table 1.

### Phylogeny of the GBSS/SSI/SSII Group

In plants, glaucophytes and green algae, enzymes of the SSIII/IV/V type have been fairly recently studied through phylogenetic analysis. These enzymes originate from a chlamydial gene encoding an enzyme secreted by these pathogens in the cytosol of the Archaeplastida common ancestor (Ball et al., 2013, 2015). Although a preliminary but incomplete analysis of the GBSSI/SSI/SSII group of enzymes has been performed, we now have investigated this issue in greater detail with a vastly expanded database that include the secondary eukaryotic algae (alveolates and cryptophytes) and a much larger sample of glaucophytes and red algae.

**FIGURE 8:**
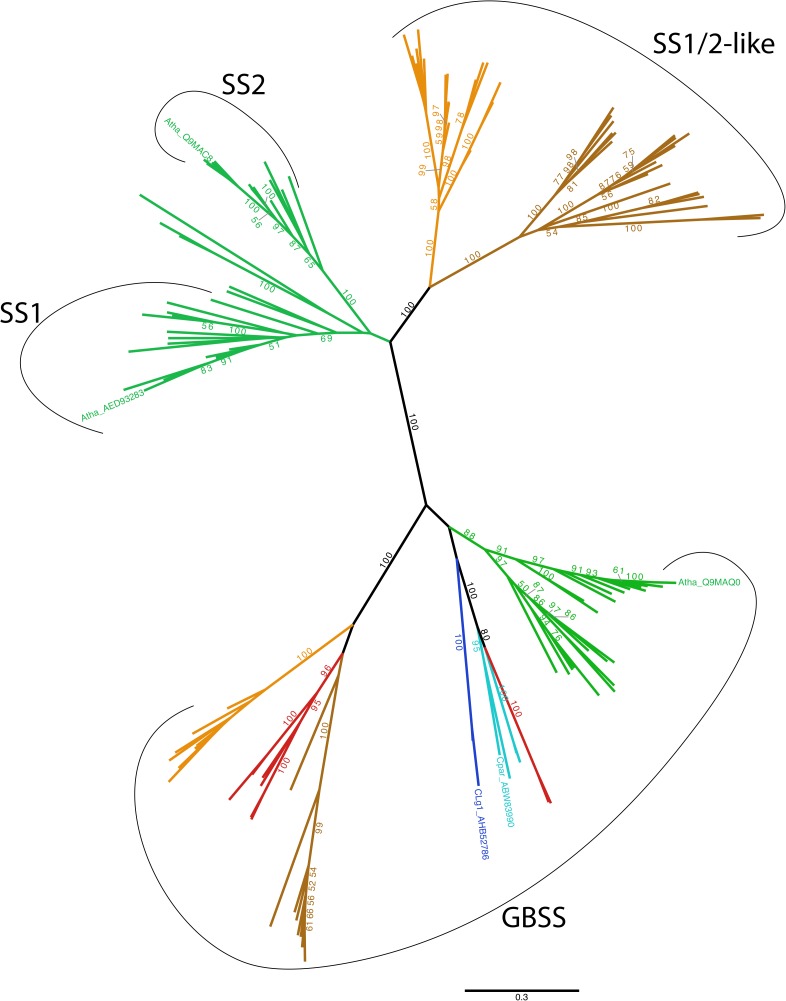
Unrooted phylogenetic tree of GBSS, SSI and SSII. The tree displayed was obtained using the LG4X model (Le et al., 2012) with IQ-TREE (Nguyen et al., 2015) and maximum likelihood bootstrap values > 50% are indicated onto the nodes. Groups are color-coded according to taxonomy: Viridiplantae are in green, Glaucophyta are in light blue, Rhodophyta are in red, Alveolata in dark brown, Cryptophyta are in light brown and Cyanobacteria are in dark blue. We specifically indicate the GBSS, SSI and SSII from *Arabidopsis thaliana* (Atha), as well as the GBSS from *Cyanobacterium* sp. CLg1 (CLg1) and *Cyanophora paradoxa* (Cpar). No other Taxa names are indicated on this unrooted tree for the sake of clarity. All clade names can be found, however, on the rooted version of this tree that is displayed in Supplementary Figure 1. The scale bar shows the inferred number of amino acid substitutions per site.

GBSS and SSI/SSII enzymes from the lineages derived from plastid primary and secondary endosymbiosis, respectively the Archaeplastida (green and red algae, glaucophytes and plants) and the alveolates and cryptophytes, form a very robust monophyletic group among the bacterial GT5 ADP-Glc requiring glycosyl transferases (**Figure 8** and Supplementary Figure 2). Outgroup rooting does not provide any additional information over midpoint rooting with respect to the source of this enzyme (Supplementary Figure 2) and many distinct gene source scenarios are possible with such topologies. This source is thus not resolved by phylogenetic analysis. However, our current knowledge of primary and secondary plastid endosymbiosis coupled to biochemical reasoning enables us to considerably restrict the number of possible scenarios (see Discussion).

Phylogenetic analysis does resolve cleanly three subgroups of sequences: these are the GBSSI-like processive enzymes found in Archaeplastida, dinoflagellates and cryptophytes; the SI/SSII group of the green algae and plants and the SSI/SSII like sequences found in the dinoflagellates (a subgroup of alveolates) and cryptophytes. The interrelations of these subgroups are discussed in the light of a cyanobacterial GBSSI source for the whole group.

## Discussion

The three new crystal structures reported greatly increase structural information on SSs. The enzymes have diverse origins (glaucophyte, cyanobacterium and plant), isoforms (GBSS and SSIV) and even donor preference (UDP-Glc and ADP-Glc). The structures presented here reveal binding modes for both acceptor and donor. The two previous SS structures either lacked (HvSSI) (Cuesta-Seijo et al., 2013) or had incomplete (OsGBSSI) (Momma and Fujimoto, 2012) ligand binding. For OsGBSSI only an ADP complex was reported with a conformation incompatible with glucose binding in the active site as its fully extended phosphates would clash with both the glucose and the acceptor sites. It can be expected that features common to these five enzymes, will be common to SS enzymes, thus helping to identify sets of fundamental and non-fundamental features in the SS family at the structural level. For analysis and comparison purposes, we will also include EcoGS, for which multiple high quality structures are available, including donor and acceptor bound structures (Sheng et al., 2009a,b). EcoGS, while not strictly an SS, has a large degree of sequence and structural similarity to SSs and utilizes ADP-Glc as donor.

### ADP Binding

ADP binding occurs through a large number of close interactions and hydrogen bonds, with most of all possible interactions utilized in the case of AtSSIV. The interactions involving the diphosphate moiety seem to be very similar in all structures, but a very different picture emerges looking at the adenine and ribose. With the exception of CpGBSSI, all structures share the sandwich interactions with the adenine, which has its pi clouds stacked against a phenylalanine or a tyrosine on one side and against the side chain of a valine or an isoleucine on the other side. Other than these, only the interaction of N6 is maintained between structures, with other nitrogen-mediated interactions moving well beyond an arbitrary 3.2 Å cutoff distance. In the ribose, O3 mediated interactions also appear to be optional, as is the water mediated one in O2. This leaves only the stacking interactions mentioned above and single hydrogen bonds from N6 and O2 to provide sufficient binding and orientation to the whole adenine-ribose assembly. Either shifts occur from the conformations captured in these crystals, possibly once the reaction has commenced, or, once ADP is bound, it might be the phosphates that provide the largest part of the energetic and electronic interactions necessary for the reaction. Interestingly, the entire set of interactions involving ADP binding to AtSSIV is present in the EcoGS structure, including mediating waters with one exception, the interaction of O2 with Tyr393. This suggests that the relaxed number of interactions in the GBSS structures might be GBSS specific. In any case, the minimal set of interactions seems to be the two sandwiching ones on the adenine, one from N6 of the adenine and one from O2 of the ribose. If we extend the analyzed set to include EcoGS, then we can include either the O2 or the O3 of the ribose. In contrast, the environment around the phosphates offers much less flexibility, with even the positions of bound waters conserved between the high quality structures of AtSSIV, CLg1GBSS and EcoGS.

Overall, the conformation of ADP in chain A of the CLg1GBSS structure and in the AtSSIV and CpGBSS structures, is similar to that observed for other retaining GTB-fold glycosyltransferases like *E. coli* Waag (PDB_ID 2iw1) (Martinez-Fleites et al., 2006), *E. coli* OtsA (PDB_ID 1uqu) (Gibson et al., 2004) and T4 bacteriophage AGT (PDB_ID 1y6f) (Larivière et al., 2005). Those structures have donor analogs with covalently bound glycosyl analogs. In those cases, also the position of these glucosyl groups could be said to be functionally equivalent to that of the amino-pyranose in the SS crystals, although significant shifts are present to account for the presence or absence of covalent bonds between the distal phosphate and the glucosyl group.

The phosphates of ADP adopt a different conformation in chain B of CLg1GBSS, with the proximal phosphate moving by 3.6 Å relative to chain A (**Figure 4**). No obvious changes are present in the environment of the protein itself, with only a minor displacement of Gly19 due to acceptor binding.

It is interesting to note that ADP in chain B of the CLg1GBSS structure adopts a conformation quite similar to that observed in structures of some GTA-fold glycosyltransferases which have donors with a covalently bound glucosyl group, for example *R. marinus* MGS (PDB_ID 2bo8 and 2y4m) (Flint et al., 2005; Nielsen et al., 2011) and the human blood group glycosyltransferase (PDB_IDs 5c3b and 5c1l) (Gagnon et al., 2015).

The fact that both conformations are present in different chains of the CLg1GBSS structure, and thus in the presence of equal solvent conditions, and even, with crystallographic disorder, simultaneously on chain C, suggests that both conformational states are possible. Nonetheless, the fact that there is perfect correlation across all structures between an extended phosphate conformation and the absence of an acceptor, whether a glucose is present in the active site or not, suggests that the extended conformation is energetically preferred. A clash would result, if both were present simultaneously, between the distal phosphate in chain B and the methyl group in acarbose in chain A, with a virtual distance of only 1.9 Å which would likely become smaller if a hydroxyl were present in that location in acarbose (**Figure 4**). It is obvious that small rearrangements would have to occur between the situation captured in these crystals, with acarbose mimicking a covalent bond already formed between the hexoses of donor and acceptor, and the situation previous to the reaction. Nevertheless, it appears that it is the presence of the acceptor itself, and likely of this C6-O6 hydroxyl group in particular, which creates steric impediments that force the phosphates in the donor to shift from the preferred extended conformation in the absence of acceptor to that observed in the presence of acceptor and which, presumably, is the one conductive to a reaction.

These observations could be extended to the structures of AtSSIV and CpGBSSI, whose acarbose molecules would clash with a hypothetical ADP molecule in the extended conformation. Comparison with the previously available structure of EcoGS also supports this notion. With HEPPSO bound to GS as an acceptor mimic (Sheng et al., 2009a), there would be a clash between the hydroxyethyl group of Heppso and the distal phosphate, with the hydroxyl forced to make two excessively short interactions simultaneously at 2.4 Å to the active site glucose and at 2.5 Å to the virtual distal phosphate of chain B in CLg1GBSS. In the structure of EcoGS with maltotriose bound in the acceptor site (Sheng et al., 2009b), the C6 carbon would be only 1.9 Å away from the virtual distal phosphate oxygen of chain B of CLg1GBSS, while it has comfortable distances of 3.7 Å to the phosphate oxygens after ADP adopts the same conformation as in chain A of CLg1GBSS. In summary, it appears that conformational selection in ADP is, in this case, under control of the acceptor molecule.

### Acceptor Binding

Acarbose binding can be separated into two different binding environments: That of the amino-pyranose moiety, which occupies the site for the glucose originating from ADP-Glc, and that of the other 3 hexoses, which occupy the sites for acceptor binding in a productive reaction.

The amino-pyranose makes the most interactions, with each hydroxyl group involved in at least two interactions either with the protein or with the phosphates of ADP. The binding mode is essentially conserved across all structures, highlighting how the conformation of this glucose and of the phosphates come to dominate the reaction. Chain B of CLg1GBSS has a glucose bound in the same site, while several EcoGS structures also feature a glucose or glucose analog there, in that case in the presence of an acceptor mimic. In all cases the conformations of those glucoses are almost perfect matches to those of amino-pyranoses in acarbose, which is thus most likely reflective of the conformation to be adopted by the glucose to be transferred during the reaction.

As for the hexoses occupying acceptor sites, only a small number of interactions seem to be maintained in all structures: In that next to the amino-pyranose, an interaction between either O2 and/or O3 and an aspartate’s side chain, with, possibly, an extra water mediated interaction of its ether oxygen, as lack of consistency in the presence of waters between different crystal structures is not proof of the absence of such waters. The glucose next to the reducing end glucose has consistent stacking interactions with aromatic side chains either side of the glucose ring in all cases, while the interactions of either O2 and/or O3 with the side chains of either a tryptophan or a histidine are also maintained with small shifts between structures. The interaction of O6 with Thr17 described for CLg1GBSS chain A, which is accompanied by a conformational change in the 15–18 loop together with ligand binding, is actually not maintained across all structures. That residue is substituted by valine in both AtSSIV and EcoGS, yet, in both cases, the loop is observed to still adopt the “bound” conformation in the presence of acceptor. It is thus not that hydrogen bond to acarbose that triggers the conformational change in the loop. The reducing end glucose is not making any interactions that are consistent between the different structures. It displays a certain degree of mobility between structures, especially if considering the maltotriose bound EcoGS structure (Sheng et al., 2009b). It is likely that the contribution of this acceptor glucose, and others potentially extending further away from the active site, will be protein specific, which is consistent with maltose being the shortest acceptor recognized by all endosperm SSs from barley (Cuesta-Seijo et al., 2016).

It can be questioned whether the conformation adopted by acarbose matches that to be expected of a bona-fide acceptor. With the exception of this glucose at the reducing end, there is very good agreement between the positions and conformation of the glucoses in the maltotriose bound to the acceptor site in EcoGS and their equivalent atoms in the acarboses described here, with distances after superposition of less than 1.1 Å in all cases except the C6 carbons and hydroxyls for the central hexoses of acarbose. Acarbose is, thus, bound in the acceptor (as well as donor) binding site in a conformation functionally equivalent to that of a natural α-glucan acceptor, deviating only by the reducing end glucosyl moiety of acarbose.

The structure of CpGBSSI is illuminating regarding selection of donor molecule. While plant SS enzymes use ADP-Glc as a donor, as does EcoGS; CpGBSSI prefers UDP-Glc as a donor instead, although it is also capable of using ADP-Glc (Plancke et al., 2008). This didn’t prevent its crystallization in the presence of ADP nor prevented ADP from being bound in the crystal structure, but the presence of adenine was merely tolerated, rather than selected for, by CpGBSSI. The adenine of ADP is found, figuratively, floating in a vacuum in the structure. This prompts the question of why is this adenine displaced from its usual binding position. As shown in **Figure 3**, the phosphates of ADP are bound similarly in this structure when compared to CLg1GBSS, with acarbose and all the surrounding loops in virtually equivalent conformations. Other than the ribose being partially rotated, likely just to accommodate the larger rotation of the adenine, the reasons for the discrepancy are to be found in the environment of the adenine itself. An isoleucine is in the same position and conformation as in the other structures, where it can be exchanged for a valine, and it would be capable of forming the same interaction if the adenine remained in place.

It is the other residue normally stacking the adenine that is responsible for the change. It is physically found occupying the space where the adenine would have been, thus that it directly prevents it from adopting its normal conformation, but the reason why it occupies that space instead of the usual stacking position next to the adenine is less clear. Many residues in its immediate environment are displaced from their positions in the other crystals, and most are also oriented differently in space, but there isn’t any single interaction that stands out as responsible for the collective movement. A proline two residues after the phenylalanine is actually found in a conformation equivalent to that of its non-proline counterparts in the other structures and thus appears not to create any extra conformational constraints. It is possible that the displacement is simply a collective phenomenon arising from the very different amino acid sequence in this area rather than having a well localized origin. The residue in position -3 is charged polar, in contrast to non-charged and mostly apolar in its five counterparts in **Figure 3**. The residue in position -2 is polar in contrast to apolar for all the other enzymes, while in position -1 there is an alanine in contrast to either a positively charged amino acid or a glycine for all others. Similarly, in position +1 there is an apolar residue in contrast to only polar residues in the homologous enzymes, and positions +4 and +5 both are glutamates in strong contrast to apolar and small polar but uncharged residues in the equivalent positions. Thus, it is possible that the whole region has simply evolved under different evolutionary constraints in such a way as not to favor the stacking interaction between that phenylalanine and the adenine. We can only speculate as to what the UDP bound conformation of CpGBSSI would look like and whether there would be any structural features selecting for it.

### Structural Relations Between Enzymes

With five different structures of SSs from different origins we can assess the level of conservation in the family at the structural level. An analysis of the position and length of secondary structural elements, also including EcoGS, as shown in **Figure 7**, shows a very high level of conservation in the order, position and length of most secondary structure elements, extending also to bacterial GSs. Analyzing sequence conservation and R.M.S.D. distances (**Table 2**), only the closer relationships in between the GBSSs and a higher relatedness of SSI to the GBSSs than to the other groups stands out. The distance, in terms of structure and sequence, between EcoGS and the GBSSs or between EcoGS and AtSSIV is no larger than the distance between the GBSSs and AtSSIV. Together with the observations already made of equivalent binding modes, it appears that there is no particular structural difference of major importance between bacterial GSs and SSs in general or plant SSs in particular.

Most of the differences to be found are in the form of extra or extra-long loops for the GBSSs, most of them corresponding to those shown in **Figure 6**. Their positions are conserved (**Figure 7**) with the exception of OsGBSSI, which uses an alternative loop 4 combined with a unique loop 3 by using the disulfide bridge to force the conformation of said loops to emulate the effect of loop 4 in CLg1GBSS or CpGBSSI, while having an altogether much smaller loop 2. The end effect of these extra loops is to surround the reducing end glucose of acarbose, imperfectly in the case of OsGBSSI but forming what resembles a closed dome around it in the case of the non-plant GBSSs. It is probable that these domes are the reason behind the processivity displayed by GBSS enzymes, by strongly binding a larger acceptor than that present in these structures at a second location other than the active site. It would be too speculative to propose here any particular protein sections as such holding sites, although analysis of the structures suggests some potential paths for the acceptor chain, some of them similar in all three GBSSs. A proper assessment of a holding site or exit channel responsible for processivity would require mutagenesis studies on these or related enzymes.

Structural studies are the best way to identify new surface binding sites for α-glucans (Cockburn and Svensson, 2013). This study identifies a new surface glucan binding site in AtSSIV and another possible site in CLg1GBSS. Both are spatially close but constitute clearly different binding sites in the respective enzymes. Their features, also at the protein level, are not replicated in any of the other SS structures. So far each of the surface binding sites identified for SSs is unique, and none of them overlap with known surface binding sites in the GSs of *E. coli* or *P. abyssi*. Thus, no surface binding sites of generic nature have been yet been found in SSs.

### Kinetic Measurements on the Enzymes in This Study

Kinetic data (Supplementary Table 1) was obtained for the purified enzymes used for crystallization as well as for a full length construct of AtSSIV purified with the same methods. AtSSIV displays an activity profile qualitatively equivalent to that measured previously for SSIV from barley (Cuesta-Seijo et al., 2016), with non-detectable activity with glucose as acceptor, low activity with maltose as acceptor and comparatively higher activities with maltodextrins as acceptors. In contrast, the measured activities were much lower with polysaccharides as acceptor molecules.

In order to increase the chances of crystallization, only the catalytic domain of AtSSIV was kept in the construct used for structural determination. A previous study on AtSSIV found that removal of a conserved dimerization region alone inhibited the activity of the enzyme when tested with amylopectin (Raynaud et al., 2016). This is not the case here, but our construct is significantly different, with removal of the whole N-terminal half of the protein, removing also the coiled-coil domains in addition to the dimerization region. While the activities measured for the crystallized construct are higher than for the full length protein in numerical terms, they are actually slightly lower when considered in a per-protein basis, but nonetheless prove that the construct used for crystallization is not impaired in its activity, at least when assayed in a simplified system. Functionality *in vivo* might differ and depend strongly on the presence of the N-terminal half of the protein (Lu et al., 2017).

CLg1GBSS, also has a similar acceptor profile to that measured for GBSSI from barley (Cuesta-Seijo et al., 2016) with the polysaccharides tested being comparatively better acceptors than maltooligosaccharides and the same behavior as AtSSIV regarding maltose and glucose. The activity with UDP-Glc as acceptor, while measurable, was several orders of magnitude lower than with ADP-Glc, and was not measurable at similar concentrations of UDP-Glc as used for ADP-Glc.

CpGBSSI was assayed with ADP-Glc as donor and a low value, in the micromolar range, was found for K_M_ for ADPG. This is unexpected in light of the crystal structure and previous reports (Plancke et al., 2008). Assays were also carried out with UDP-Glc as donor. At concentrations comparable to those used for ADP-Glc, no measurable activity was present. Increasing the concentration of UDP-Glc to 100 mM resulted in measurable activity but no steady state conditions, preventing us from quantifying it. In any case, the activities would, as for CLg1GBSS, be more than an order of magnitude smaller than with ADP-Glc at biologically relevant conditions. The reasons for these discrepancies are unknown, but it must be mentioned that the assays performed in this study are done in solution with purified protein, in contrast with proteins embedded inside starch granules in Plancke et al. (2008), which is the natural environment for a GBSS enzyme.

### Origin of the GBSS/SSI/SSII Group

Biochemical reasoning coupled to our knowledge of primary endosymbiosis suggests that a cyanobacterial source constitutes the most likely scenario for defining the source of the whole group of GBSS/SSI/SSII among the bacterial GT5 glucan synthases. Indeed, Archaeplastida and some of their secondary endosymbiosis derivatives are the only eukaryotes encoding such GT5 glycosyl transferases. We can thus logically assume a bacterial source for this enzyme, restricting the number of possible scenarios to two. First, GBSSI could have evolved within cyanobacteria or second, GBSSI could have evolved within the bacterial outgroup displayed in Supplementary Figure 2 while cyanobacteria would have received the gene by lateral gene transfer (LGT) from a eukaryotic alga. We argue that if the few cyanobacteria (only two sequences in the databases) that harbor GBSSI had received the gene from a eukaryotic source, these bacterial sequences should have been found within either one of the three Archaeplastida groups or within a secondary endosymbiosis group such as the alveolates or cryptophytes. This is not the case. The phylogenetic position and monophyletic nature of cyanobacterial GBSSI at the base of the three Archaeplastida lineages, is, on the contrary, perfectly consistent with the gene coding this enzyme entering the ancestral eukaryotic host through primary endosymbiosis.

Because of this, and in agreement with our previous biochemical characterization of cyanobacterial GBSS (Deschamps et al., 2007), we propose that the cyanobacterial plastid ancestor was a starch and amylose accumulating cyanobacterium. Most cyanobacteria are glycogen accumulators and only a few groups (six) have been demonstrated to synthesize starch-like material and fewer yet (two) to contain amylose (reviewed in Ball et al., 2015). It will thus be of interest to further explore the diversity of basal Gloeobacterales (inclusive of *Gloeomargarita lithophora*) suspected to be, among extant cyanobacteria, the closest relatives to the plastid donor (Ponce-Toledo et al., 2017).

The cyanobacterial GBSSI subsequently evolved to yield the green and red alga as well as the glaucophyte GBSSI activities. This was accompanied by a less restrictive substrate preference, as all these enzymes, unlike the cyanobacterial extant enzymes, are nowadays able to use both UDP-Glc and ADP-Glc (Plancke et al., 2008; Price et al., 2012; Bhattacharya et al., 2013). In the red algae, the GBSSI gene was lost twice: upon diversification of the thermophilic cyadiniophytina on the one hand, and upon evolution of the multicellular mesophilic red algae on the other hand. Interestingly, these losses correlate with those of a pyrenoid based carbon-concentrating mechanism in these red algae. We have indeed suggested previously that the tight physical association of starch with the pyrenoid required the presence of amylose (Ball et al., 2011; Izumo et al., 2011). The mesophilic red algae in turn were recruited to generate the secondary plastids, thereby explaining both, on the one hand the presence of red alga-affiliated GBSSI and amylose in cryptophytes and dinoflagellates (Deschamps et al., 2006, 2008a; Curtis et al., 2012) and on the other hand the attraction of a subset of mesophilic unicellular red alga sequences within the secondary endosymbiosis lineages. Interestingly the green algae and plant GBSSI sequences display a sister rather than the expected nested topology with the reminder Archaeplastida and cyanobacteria. This is also seen in the case of other enzymes of starch metabolism such as GlgX/ISA1,2,3 (debranching enzyme) or GlgA2/SSIII/IV (Ball et al., 2013) where the rewiring of the starch synthesis network from the cytosol to the chloroplast led to sequence evolution acceleration and phylogenetic signal erosion. Note that the sisterhood topology is in all cases weakly supported (BV 52 or BV 38 when including the bacterial outgroup, Supplementary Figure 2).

We have previously outlined the reasons suggesting that the common ancestor of the Archaeplastida displayed synthesis of storage polysaccharides in the cytosol where glycogen/starch synthesis occurred from both UDP-Glc and ADP-Glc (Deschamps et al., 2007). The loss of cyanobacterial starch synthesis by the evolving plastid was accompanied by that of all plastidial cyanobacterial enzymes with the exception of ADP-glucose pyrophosphorylase (Deschamps et al., 2007). During the evolution of the green algae, a strong selection favored the return of storage polysaccharide to the chloroplast (Deschamps et al., 2008b,c). This return was technically difficult to achieve due to the prior loss of all genes encoding enzymes of storage polysaccharide synthesis from the plastome. It entailed three stages yielding respectively malto-oligosaccharides, glycogen and finally plastidial starch which was accompanied by loss of cytosolic starch leaving the amylomaltase Dpe2, cytosolic phosphorylase and possibly the heteroglycan pool as remnants of the cytosolic storage polysaccharide pathway (Deschamps et al., 2008b; Ball et al., 2011). Several rounds of gene duplication and enzyme subfunctionalisation occurred, yielding the complexity of starch metabolism that explains the highly redundant pathway witnessed today selectively in both the green algae and land plants. The cytosolic glycogen/SSs of cyanobacterial (GBSS) and chlamydial (SSIII/IV) origins experienced several rounds of gene duplications yielding respectively plastidial GBSSI, SSI and SSII (Supplementary Figure 2) and plastidial SSIII/IV/V (Ball et al., 2013).

Red algae and glaucophytes as well as many heterotrophic eukaryotes carry another glucosyl transferase enzyme of eukaryotic ancestry with a substrate preference restricted to UDP-Glc (reviewed in Ball et al., 2011, 2015). This enzyme of CAZy family GT5 is as distantly related to the bacterial GT5 ADP-Glc requiring glucosyl transferases as the latter are to the well-studied UDP-Glc specific GT3 glucosyl transferases which define the second type of glycogen synthases encoded by eukaryotes (reviewed in Ball et al., 2015). All glaucophytes and red algae encode this ancient GT5 transferase enzyme of eukaryotic glycogen metabolism (Ball et al., 2015). Remarkably a single Swiss knife enzyme of this type is sufficient to achieve starch synthesis including synthesis priming and elongation of different glucan sizes which otherwise requires a minimum of five enzymes in green algae and plants. We have previously detailed the reasons why this enzyme could not be maintained through secondary endosymbiosis in specific lineages such as the cryptophytes (Curtis et al., 2012). Briefly in these algae, natural selection has favored the maintenance of starch metabolism in the periplast compartment (Deschamps et al., 2006) which corresponds to the ancient red alga cytosol between the second and third membrane of the four-membrane secondary plastids. Because we believe the GT5 UDP-Glc transferase does not have the built-in capacity to interact with chaperones and be readily targeted to this compartment, recruitment of a gene from a foreign organism that fulfilled these requirements happened faster. A green alga SSI/II gene that already carried a transit peptide was selected though LGT for its ability to be efficiently targeted to plastids (including the periplast) and was more rapidly recruited (Curtis et al., 2012; Ball et al., 2015). Recruitment by LGT from the environment of green alga genes seems indeed to be common theme in secondary endosymbiosis involving red alga endosymbionts. The green alga sequence accumulated mutations to optimize its activity with respect to UDP-Glc and was duplicated and subfunctionalized to progressively take over the multiple functions of the Swiss-knife rhodophycean enzyme (Curtis et al., 2012). This generated the multiple SSI/SSII like enzymes of dinoflagellates and cryptophytes evidenced in Supplementary Figure 2. Finally the tree topology of the SSI/SSII-like UDP-Glc specific enzymes of secondary algae is compatible with many different scenarios. Independent recruitment in dinoflagellates and cryptophytes of a related SSI/SSII source as well as LGT from either dinoflagellates to cryptophytes (or vice versa) are equally viable hypotheses.

In summary, the new SS structures presented here, expanding beyond the plant kingdom, illustrate and confirm the mode of binding of donor and acceptor in a ternary complex with SS. We defined the minimum and variable set of interactions between the protein and either the donor or the acceptor which are present in all structures determined so far. We identified loop movements in response to acceptor binding and structural elements likely responsible for the processivity of GBSSs. We also reported a detailed evolutionary history for GBSSs and the related SSI and SSII isozymes.

## Data Availability Statement

The datasets, coordinates and structure factors, corresponding to the crystals presented in this study can be found in the Protein Data Bank https://www.rcsb.org/.

## Author Contributions

MN, CR, SB, MP, and JC-S conceived and designed the experiments. MN, CR, KK, AS, and JC-S prepared the materials and performed the experiments. UC and SB conceived and provided the phylogenetic analysis. MN, CR, AS, UC, SB, MP, and JC-S contributed to the writing of the paper.

## Conflict of Interest Statement

The authors declare that the research was conducted in the absence of any commercial or financial relationships that could be construed as a potential conflict of interest.

## Footnotes

1 www.pymol.org
