# Supplementary material for: Crystal Structures of the Catalytic Domain of Arabidopsis thaliana Starch Synthase IV, of Granule Bound Starch Synthase From CLg1 and of Granule Bound Starch Synthase I of Cyanophora paradoxa Illustrate Substrate Recognition in Starch Synthases
Source: Front Plant Sci. 2018 Aug 3;9:1138. doi: 10.3389/fpls.2018.01138 (PMC6086201; doi:10.3389/fpls.2018.01138)
Supplement: Supplementary file 1 [file Table_1.DOCX]

|  | AtSSIV | AtSSIV_CD | CLg1_GBSS | Cp_GBSSI |
| --- | --- | --- | --- | --- |
| 100mM Glc1, 1 mM ADPG | < 0 | < 0 | < 0 (10 mM) |  |
| 10 mM Glc2, 1 mM ADPG | 3.48 | 5.19 | 0.0014 |  |
| 10 mM Glc3, 1 mM ADPG | 19.81 | 34.78 | 0.0143 |  |
| 10 mM Glc4, 1 mM ADPG | 17.39 | 33.74 | 0.0396 |  |
| 10 mM Glc5, 1 mM ADPG | 15.13 | 23.54 | 0.0298 |  |
| 10 mM Glc6, 1 mM ADPG | 16.31 | 26.49 | 0.0123 |  |
| 10 mM Glc7, 1 mM ADPG | 15.13 | 26.27 | 0.0168 |  |
| 10 mM Glc8, 1 mM ADPG | 16.31 | 18.33 | 0.0174 |  |
| Soluble Potato starch 1 mg/mL, 1 mM ADPG | 0.074 | 0.11 | 0.2460 |  |
| Glycogen 1 mg/mL, 1 mM ADPG | 0.27 | 0.03 | 0.0321 |  |
| Amylopectin, maize, 1 mg/ml, 1 mM ADPG | 0.72 | 0.56 | 0.0717 |  |
| K_M_ for ADPG (1 mg/mL glycogen) |  |  | 0.46±10 mM |  |
| K_M_ for ADPG (10 mM Glc3) |  |  | 0.40±8 mM |  |
| 100 mM UDPG, 10 mM Glc3 |  |  | 0.00032 |  |
| 0.5 mM UDPG, 1 mg/mL glycogen |  |  |  | Non-detectable |
| 0.5 mM ADPG, 1 mg/mL glycogen |  |  |  | 0.0908 |
| 0.5 mM ADPG, 1 mg/mL glycogen |  |  |  | 0.1038 |
| K_M_ ADPG (1 mg/mL glycogen) |  |  |  | 0.083±11 mM |
| K_M_ for glycogen (0.5 mM ADPG) |  |  |  | 0.312±15 mM |
| K_cat_ with glycogen (0.5 mM ADPG) |  |  |  | 0.1123±10 mg/mL |
