# Supplementary material for: Crystal Structures of the Catalytic Domain of Arabidopsis thaliana Starch Synthase IV, of Granule Bound Starch Synthase From CLg1 and of Granule Bound Starch Synthase I of Cyanophora paradoxa Illustrate Substrate Recognition in Starch Synthases
Source: Front Plant Sci. 2018 Aug 3;9:1138. doi: 10.3389/fpls.2018.01138 (PMC6086201; doi:10.3389/fpls.2018.01138)
Supplement: Supplementary file 2 [file Data_Sheet_1.pdf]

frontiers

Supplementary Material

Crystal Structures of the catalytic domain of *Arabidopsis thaliana* Starch Synthase IV, of Granule Bound Starch Synthase From CLg1 and of Granule Bound Starch Synthase I of *Cyanophora paradoxa* Illustrate Substrate Recognition in Starch Synthases

Morten M. Nielsen, Christian Ruzanski, Katarzyna Krucewicz, Alexander Striebeck, Ugo Cenci, Steven G. Ball, Monica M. Palcic, Jose A. Cuesta-Seijo*

*** Correspondence:** Jose A. Cuesta-Seijo: josea.cuesta.seijo@carlsberg.com

1 Supplementary Data

Protein sequences expressed:

```
CLg1GBSS:
MGSSHHHHHHSSGLVPRGSHMLNICFVSTEVAPYSKTGGLGDVTEGLPEELAKIGHKVCT
VAPRFDQYEDAWDTEIIQPVNYGQEKTNVRYFHSYKKGVDHIWVDHHVYLSKTPLVNKKL
YGPKDSVDYIDNVERFAMLSQAALAVPLLVPLGAKGSQGVMGENTIFVCNDWHTSLLPLY
LKEYYQSQGIFVNAKTVMLLHNIAFQGRFPSSKFDALNLPAKYLSDLSFNTQFAPPPLDE
KTTEPITSPEPMYMLNWLKAGFLNCDQALTVSPNFAHEVTSSPMGGVELDAVARDVGLTG
ITNGTKIETWNPQKDKFILANYNSRTINSGKKLCKVALQKECGLTVDPDIPLFGFIGRLE
NQKGADVIIAAMPKLKQLNCQVVILGIGSPKLEQELESVADKYPFAKGVARFDSKLAHFI
TAGADYCLMPSRFEPCGLNQLYAMMYGTIPVVAPVGGLVDTVPPQFGFLMNKIPMPKIPG
VTVSEELLQQGVDAMIVGMKKALQEYGTPKFKKMRLDCMANDVSWKKPAAKYVDIFEQLV
NSQV

Catalytic domain of AtSSIV for crystallization:
LVSSPTSSGLYVVHIAAEMAPVAKVGGLGDVVAGLGKALQRKGHLVEIILPKYDCMQYDRVRDLRAL
DTVVESYFDGKLYKNKIWIGTVEGLPVHFIEPQHPSKFFWRGQFYGEQDDFRRFSYFSRAALELLLQ
SGKKPDIIHCHDWQTAFVAPLYWDLYAPKGLDSARICFTCHNFEYQGTASASELGSCGLDVNQLNRP
DRMQDHSSGDRVNPVKGAIIFSNIVTTVSPTYAQEVRTAEGGKGLHSTLNFHSKKFIGILNGIDTDS
WNPATDPFLKAQFNAKDLQGKEENKHALRKQLGLSSAESRRPLVGCITRLVPQKGVHLIRHAIYRTL
ELGGQFVLLGSSPVPHIQREFEGIEQQFKSHDHVRLLLKYDEALSHTIYAASDLFIIPSIFEPCGLT
QMIAMRYGSIPIARKTGGLNDSVFDIDDDTIPTQFQNGFTFQTADEQGFNYALERAFNHYKKDEEKW
MRLVEKVMSIDFSWGSSATQYEELYTRSVSRARAVPNRT
```

A C-terminal IDPFTENLYFQGHHHHHH sequence was included in the expressed construct and cleaved with TEV protease during purification.

```
Full length AtSSIV for activity assays:
```

MCKMRQQRGFDSSKRQEVKKGSPKPILSINSGLQSNNDEESDLENGSADSVPSLKSDAEKGSSIHGS
IDMNHADENLEKKDDIQTTEVTRRKSKTAKKKGESIHATIDIGHDDGKNLDNITVPEVAKALSLNKS
EGEQISDGQFGELMTMIRSAEKNILRLDEARATALDDLNKILSDKEALQGEINVLEMKLSETDERIK
TAAQEKAHVELLEEQLEKLRHEMISPIESDGYVLALSKELETLKLENLSLRNDIEMLKSELDSVKDT
GERVVVLEKECSGLESSVKDLESKLSVSQEDVSQLSTLKIECTDLWAKVETLQLLLDRATKQAEQAV
IVLQQNQDLRNKVDKIEESLKEANVYKESSEKIQQYNELMQHKVTLLEERLEKSDAEIFSYVQLYQE
SIKEFQETLESLKEESKKKSRDEPVDDMPWDYWSRLLLTVDGWLLEKKIASNDADLLRDMVWKKDRR
IHDTYIDVKDKNERDAISAFLKLVSSPTSSGLYVVHIAAEMAPVAKVGGLGDVVAGLGKALQRKGHL
VEIILPKYDCMQYDRVRDLRALDTVVESYFDGKLYKNKIWIGTVEGLPVHFIEPQHPSKFFWRGQFY
GEQDDFRRFSYFSRAALELLLQSGKKPDIIHCHDWQTAFVAPLYWDLYAPKGLDSARICFTCHNFEY
QGTASASELGSCGLDVNQLNRPDRMQDHSSGDRVNPVKGAIIFSNIVTTVSPTYAQEVRTAEGGKGL
HSTLNFHSKKFIGILNGIDTDSWNPATDPFLKAQFNAKDLQGKEENKHALRKQLGLSSAESRRPLVG
CITRLVPQKGVHLIRHAIYRTLELGGQFVLLGSSPVPHIQREFEGIEQQFKSHDHVRLLLKYDEALS
HTIYAASDLFIIPSIFEPCGLTQMIAMRYGSIPIARKTGGLNDSVFDIDDDTIPTQFQNGFTFQTAD
EQGFNYALERAFNHYKKDEEKWMRLVEKVMSIDFSWGSSATQYEELYTRSVSRARAVPNRT

A C-terminal IDPFTENLYFQGHHHHHH sequence was included in the expressed construct and cleaved with TEV protease during purification.

CpGBSSI:

MGSSHHHHHHSSGLVPRGSHMNIAPVSELQAAIDQAEKKLTIVFVGSECTPWSKTGGLGDVMRDLPV
NLAQRGHRVMSIQPRYDQYFDAWDTAVRSSIKVNGKLEDVGFFHITSKGVDRIFIDHPWFLAKVWGI
TGNKLYGAKTGVDYPDNPMRFALMCQAALEAPLRIPLPDPAGTVYGEDVIFVCNDWHSALVPIYLKA
NYKTRGLYQNAKSIFLLHNIIYQGRFPLEFWPALNLPEAAKKDLVFESCFAPPPLDGISEQPIISLK
PMAMMNFLQAGFIHADRICTVSPQFAAEVASGPRGGVELDKYIRAKGITGIMNGMDIEMWDASKDKF
LVTKYTASSVDEGKAANKAVLQAEMGLKVSPTTPLIAFVGRLDDQKGADCMVEAMPYLVNTLGAQVV
CYGSGREDMAAKFKALEKQFPGMAKGKTAFVPKEEHTLMAGADYVLMPSRFEPCGLVQLHAMKYGAV
PIVSCTGGLKDSVIPECGFTFEEIPSPEYPGMKISPELIAKGTKIIEEGCKEALAGYGSKAFAGMRA
ACMKQDFAWKKRVLVYEKVFYETLGIDRGAPVTAKASPVAPEPSAAPAPATISAATSGGILPVPKAA
APKAPKVGA

2 Supplementary Figures and Tables

For more information on Supplementary Material and for details on the different file types accepted, please see here.

2.1 Supplementary Figures

Supplementary Figure 1.

Further details in the structure of AtSSIV. The protein is shown as sticks with green carbons; ligands are shown as ball and stick with gray carbons. A) Details around His190. Yellow dashed lines highlight its proximity to the anomeric carbon of a hypothetical glucose and its orientation relative to the distal phosphate of ADP. B) Conformation of the surface binding site formed by Trp172 and Phe237, with the modelled maltose shown.

Supplementary Figure 2.

Phylogenetic tree of GBSS and SSI and SSII rooted with an outgroup (A) or focusing on algae synthase with midpoint rooting (B). The trees were obtained with IQ-TREE (Nguyen et al., 2015)

and maximum likelihood bootstrap values from 100 repetitions are indicated onto the nodes, only nodes > 50% are shown. Groups are color-coded according to taxonomy: Viridiplantae are in green, Glaucophyta are in light blue, Rhodophyta are in red, Alveolata in dark brown, Cryptophyta are in light brown, Cyanobacteria are in dark blue, and other bacteria are in black. The scale bar shows the inferred number of amino acid substitutions per site. The tree in A is manually rooted while tree in B is midpoint rooted. Because GT5 ADP-Glc using glycogen synthases are not found in eukaryotes with the exception of Archaeplastida, it is reasonable to hypothesize a prokaryotic source for this enzyme, as discussed in the text, leaving only two candidate bacterial taxa for the tree's true root. These are highlighted by grey arrows in panels A and B.

Supplementary Table 1.

Specific activities measured for the enzymes in this study. Activities were measured chromogenically as described in (Cuesta-Seijo et al., 2016) and are given in µmol of product per minute and per mg of protein. Glc1 to Glc8 represent the series of maltodextrins from glucose to maltooctaose. For AtSSIV, there are two columns of data: “AtSSIV” for the full length protein, and “AtSSIV_CD” for the catalytic domain construct used for crystallization. CLg1GBSS was non-saturable with either glycogen or Glc4, hence no K_{cat} values are given. The concentrations of both donor (ADP-glucose or UDP-glucose) and acceptor used in each assay are given in the first column.
