# Supplementary figures and images for: Crystal Structures of the Catalytic Domain of Arabidopsis thaliana Starch Synthase IV, of Granule Bound Starch Synthase From CLg1 and of Granule Bound Starch Synthase I of Cyanophora paradoxa Illustrate Substrate Recognition in Starch Synthases
Source: Front Plant Sci. 2018 Aug 3;9:1138. doi: 10.3389/fpls.2018.01138 (PMC6086201; doi:10.3389/fpls.2018.01138)

A

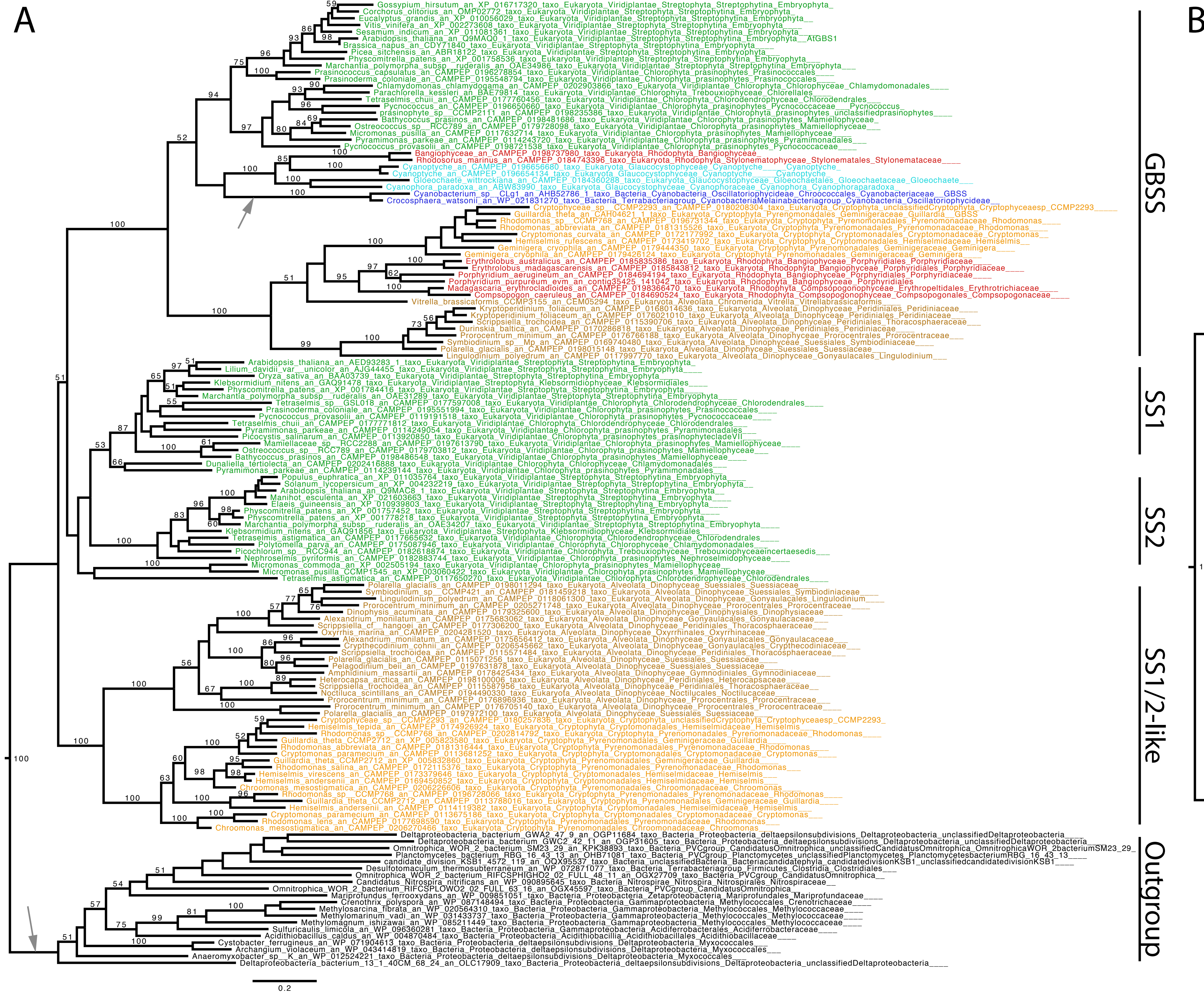

B

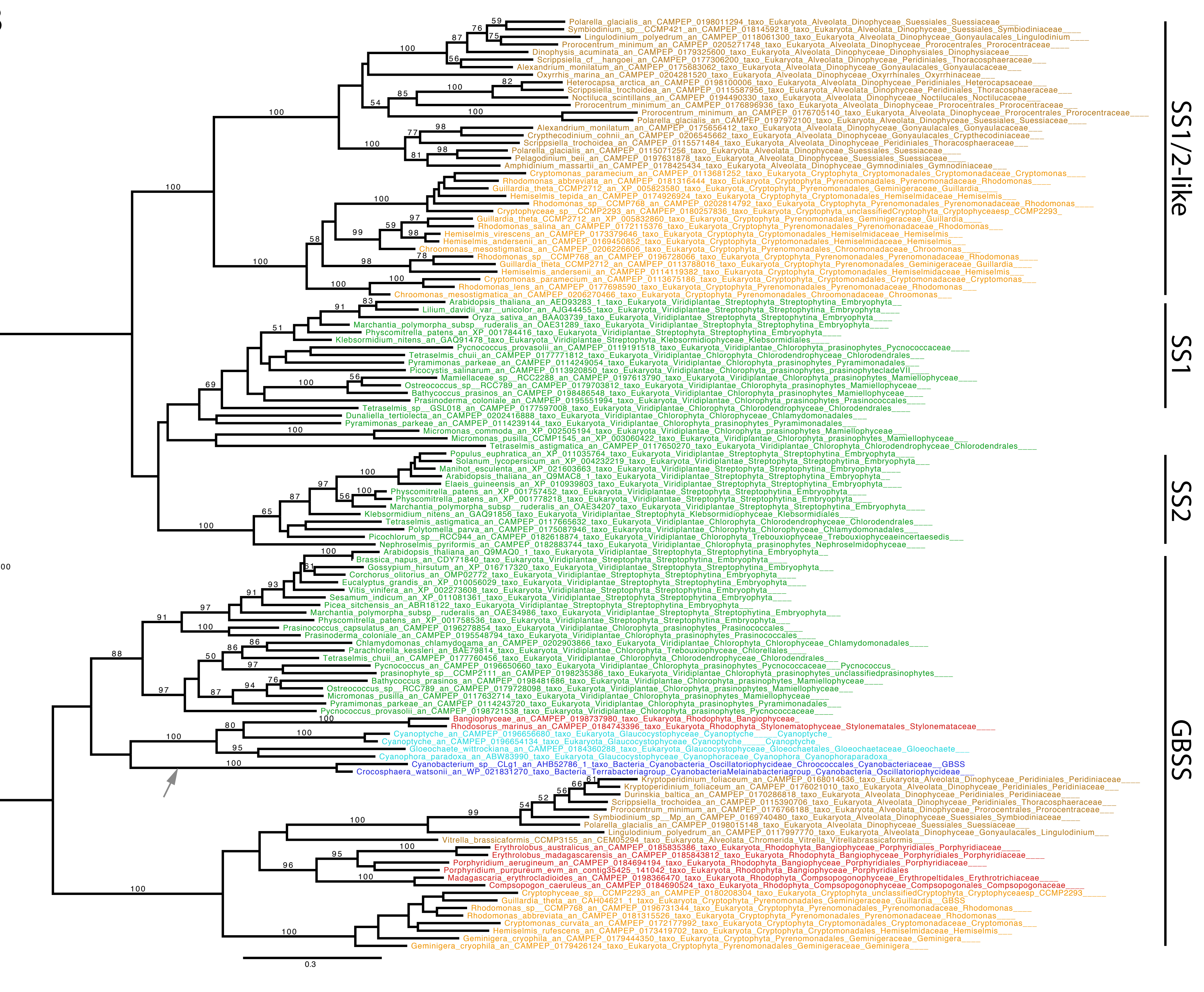

Supplement: Supplementary file 3 [file Image_2.pdf]

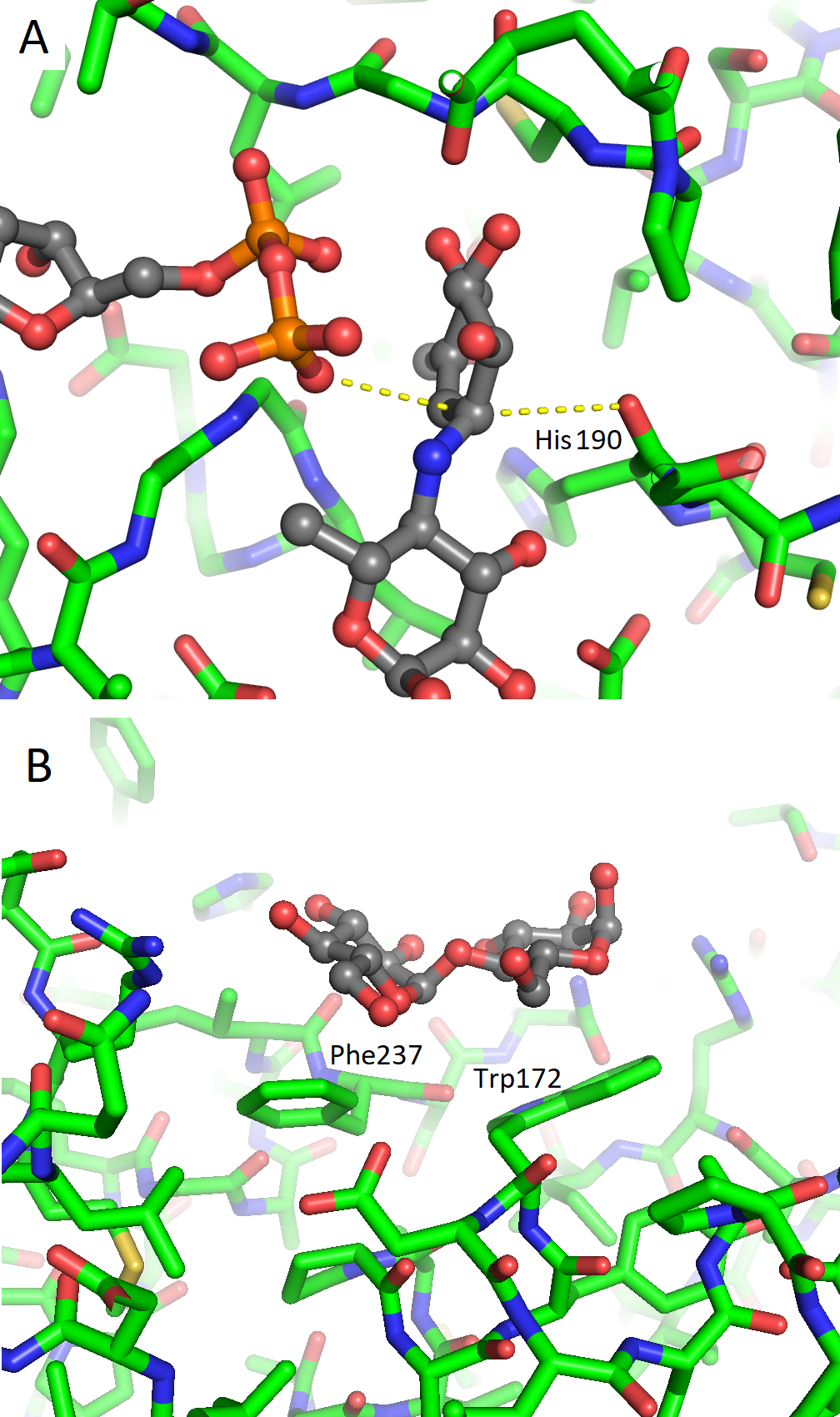

Supplement: Supplementary file 4 [file Image_1.TIF]
